# Simultaneous measurement of target and effector cell death, apoptosis, and proliferation in cell therapy development using flow cytometry

**DOI:** 10.1016/j.xpro.2026.104703

**Published:** 2026-07-23

**Authors:** Kristian Thomson, Kendra Klatt, Niklas Engels, Camille Dourlens, Daniel Schäfer, Melissa Quadflieg, Hannah Johanssen, Raphael Koch, Jürgen Wienands, Gerald Wulf

**Affiliations:** 1 Clinic for Hematology and Medical Oncology, University Medical Center Göttingen, Göttingen, Lower Saxony 37075, Germany; 2 Institute for Cellular and Molecular Immunology, University Medical Center Göttingen, Göttingen, Lower Saxony 37073, Germany; 3 Miltenyi Biotec B.V. & Co. KG, Bergisch Gladbach, North Rhine-Westphalia 51429, Germany; 4 Translational Molecular Imaging Group, Max-Planck Institute for Multidisciplinary Sciences, Göttingen, Lower Saxony 37075, Germany

**Keywords:** Cell Biology, Cancer, Health Sciences, High Throughput Screening, Immunology

## Abstract

The development of cellular immunotherapies against cancer requires an efficient assessment of malignant target cell killing. However, the fate of effector cells is equally important, particularly in the context of immunomodulatory tumors. Here, we present a high-throughput protocol for simultaneously measuring viability, apoptosis, and proliferation of cytotoxic effector cells and their targets. We describe steps for co-culturing target and effector cells, staining procedures for viability and apoptosis, adding fluorescent counting beads for cell quantification, and procedures for flow-cytometric analysis.

## Highlights

• Protocol for target and effector cell preparation and co-culture
• Instructions for the detection of cell death, apoptosis, and absolute cell number
• Guidance for flow-cytometric acquisition and data analysis

## Abstract (editor highlights)

Publisher’s note: Undertaking any experimental protocol requires adherence to local institutional guidelines for laboratory safety and ethics.

## Graphical abstract

**Figure:**
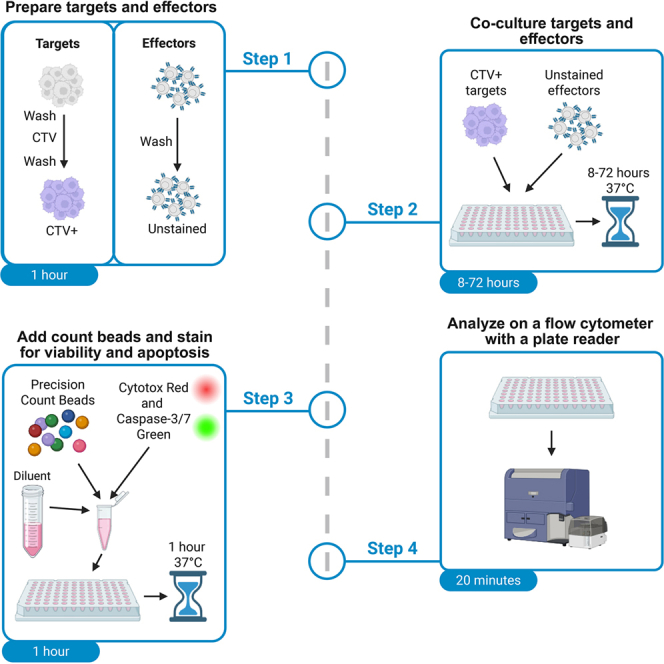

## Before you begin

This protocol details the procedure for performing a cytotoxicity assay to simultaneously assess the viability, apoptosis, and proliferation of both target and effector cells. The protocol is straightforward, fast, robust, cost-effective, and amenable to high-throughput analyses. It does not require the generation of fluorescent or luminescent reporter target cells. The end user can gather population-level and single-cell-level data on the frequency of dead and dying target cells and the absolute number of remaining healthy cells. Crucially, the impact of the target-effector interaction on effector cells can be quantified. This provides insights into effector cell fitness and enables the identification of activation-induced cell death (AICD) and potential reciprocal cytotoxic activity by target cells. The latter point is especially important in the context of malignancies with cytotoxic phenotypes. Here, we demonstrate the capacity of this protocol to measure the cytotoxic potential of CAR-T cells, γδ T cells, NK cells, CAR-NK cells, and NK92 cells. However, the main principles and procedures can be employed to evaluate the cytotoxicity of other cellular or non-cellular treatments against target cells grown in suspension. Access to tissue culture incubators, a class II biological safety cabinet, a benchtop centrifuge, and a flow cytometer with violet, blue, and red lasers is essential. For automated 96-well plate analysis, the cytometer must be equipped with a plate reader; otherwise, samples can be transferred to tubes and loaded manually.

### Innovation

This protocol leverages several well-established methods and technologies and combines them into a single straightforward, robust workflow. Commercially available non-toxic amine-reactive dyes are used to label target cells at the beginning of the protocol. Cell death is detected with cell membrane-impermeable, non-toxic DNA stains, while apoptosis is measured using activated caspase-3/7-sensitive dyes. Target and effector cell numbers are quantified using commercially available fluorescent counting beads after a co-culture of up to 72 h. End-point stains and counting beads are combined in an easy-to-use master-mix that is applied directly to the assay plate wells. The endpoint staining protocol requires no tedious, error-prone wash or centrifugation steps. Data are recorded on a flow cytometer equipped with a 96-well plate loader. Automated quality control is used to ensure data integrity before analysis. The combination of these techniques enables rapid, cost-effective *in vitro* screening of several key parameters critical to the performance of novel cellular immunotherapies.

Many different methods are available for quantifying cell cytotoxicity, each with distinct advantages and disadvantages.^1^^,^^2^ The protocol outlined here improves on several disadvantages of other protocols and combines previously separated readouts into a single assay. The current protocol does not rely on cellular metabolism, which varies significantly between cells,^3^ and may be altered for reasons other than cell death.^4^^,^^5^ Assays that rely on the reduction of metabolic activity may be unable to distinguish between cytotoxic and cytostatic effects.^2^ Colorimetric assays are simple and cost-effective. However, fluorescent methods are typically more sensitive^6^ and are easier to multiplex. Furthermore, the current protocol does not use radioactive materials, thereby eliminating the potential for user exposure to radioactivity.

Live cell imaging is an excellent option for cytotoxicity assays and offers several advantages over traditional methods. These include visualization of target-effector cell interactions, ease of quantifying cell death over time, fluorescent readouts, and multiplexing. However, there are several drawbacks. Live cell imaging requires specialized equipment and, while many life science research groups have access to flow cytometers, fewer labs own live cell imagers. Live cell imaging requires hundreds of images to be captured for accurate analysis and, therefore, requires more storage space than flow cytometry data. As such, the processing of data generated by the current protocol is less time- and computationally intensive. Overlapping cells and clumps confound microscopic analysis but are disrupted when pipetting and during flow cytometry acquisition in the current protocol. This mitigates the negative impact of cell clusters regularly formed by activated immune cells. The photomultiplier tubes (PMTs) of a flow cytometer enable the detection of exceptionally dim fluorescent signals. This allows the titration of reagents to very low concentrations, leading to more cost-effective protocols than those achieved with live cell imaging. Repeated exposure to fluorescent light on live imaging equipment can result in significant photobleaching or phototoxicity. Lastly, live cell imaging for cytotoxicity assays may require tens of thousands of cells per well to enable accurate analysis, making it unsuitable for rare cells.

The presented protocol enables simultaneous measurement of both target *and* effector cell viability, apoptosis, and absolute cell number. This is carried out in a straightforward, cost-effective, high-throughput format suitable for samples with low cell numbers.

### Institutional permissions

Production of CAR-T and CAR-NK cells often requires the transduction of cells with viral vectors. Manipulations involving infectious, replication-incompetent retroviral or lentiviral particles were carried out in biosafety level 2 laboratories in accordance with institute, state, and national regulations. Only appropriately trained and approved staff were allowed to work with virus particles. Biological wastes were discarded according to institute, state, and national level requirements.

For the isolation of T cells and the production of CAR-T cells, PBMCs of anonymized, healthy donors were separated from erythrocyte/platelet-depleted blood samples. These were collected from residual materials from blood donations that would otherwise have been discarded. Healthy donor material was provided by the Central Department of Transfusion Medicine of the University Medical Center, Göttingen, after approval by the Ethics Commission of the University Medical Center, Göttingen (application number: 3/9/21). For the isolation of NK cells and the production of CAR-NK cells from healthy donor PBMCs, materials were obtained from anonymous donors after written informed consent was given, as approved by the ethics committee of Ärztekammer Nordrhein (2020272).

### Generation of target and effector cells

**Timing: Variable (days to weeks)**

Target and effector cells must be generated before using the current protocol. For the assessment of specific killing, target cells grown in suspension expressing the target antigen are required. Cells that do not express the target antigen may be used as antigen specificity controls. Jurkat cells are a T cell line derived from a patient with T cell acute lymphoblastic leukemia (T-ALL). They express no specific antigen targeted in the current work, but are sensitive to the innate cytotoxic activity of NK cells and γδ T cells. Loucy cells are also a T cell line derived from a patient with T-ALL. However, these cells demonstrate a slight increase in resistance to killing by γδ T cells. Ramos cells are a B cell line derived from a patient with Burkitt’s lymphoma. They are CD19-positive and make up the antigen-specific target in the current work.

For CAR-T, CAR-NK, or TCR-T cells, it is important to establish transgene receptor expression. Wild-type, antigen specificity, and truncated CAR controls may be included as negative-control effector cells. NK92 cells are an IL-2-dependent natural killer cell line derived from a patient with non-Hodgkin lymphoma. These cells demonstrate robust innate cytotoxic abilities and can be redirected by CARs. The CAR-T and CAR-NK cells used in this work were derived from healthy donors and expressed anti-CD19 CARs. Upon CAR engagement with target antigens, cells become activated and may kill target cells. γδ T cells are a rare unconventional population of T cells that have an innate capacity for malignant cell killing. They were derived from healthy donors in the current work and were not transduced to express CARs.

***Note:*** The timing of target and effector cell generation will vary significantly depending on the cells required. Cell line-based targets that express the antigen of interest are ready immediately. Similarly, WT effector cells (e.g., NK92) are ready once they are healthy and at the appropriate density. Wild-type T, NK, or γδ T cells may be ready within days. However, transgene-expressing target cells, CAR-T cells, CAR-NK cells, and CAR-NK92 cells may take weeks to produce.

1. Generate target and effector cells.
  a. Ensure that target and effector cells have the appropriate characteristics (e.g., target cells express the antigen of interest, effector cells have adequate CAR expression, etc.)
  b. Culture cells to an appropriate density in the appropriate media volume 24 h prior to the start of the assay.

***Note:*** We routinely keep γδ T cells, CAR-T cells, NK cells, and CAR-NK cells at approximately 1 × 10^6^ cells/mL. We routinely keep Jurkat and NK92 cells at 4 × 10^5^ cells/mL, Ramos cells at 6 × 10^5^ cells/mL, and Loucy cells at 7 × 10^5^ cells/mL.

***Note:*** The appropriate media volume will depend on the assay size, i.e., the number of different conditions to be assessed.

***Note:*** The media for each cell type used in the examples in the current manuscript are listed in the ‘Materials and equipment’ section.

***Note:*** To save time and resources, cell density and health are routinely checked visually. However, cells can be counted with trypan blue and a hemocytometer to ensure adequate cell numbers and high viability.

**CRITICAL:** For an accurate cytotoxicity assay, cells must be healthy and at an appropriate density before beginning the protocol. If the cells are too dense or the media is too acidic, the cells may be unhealthy and yield inaccurate results and should be diluted to an appropriate cell concentration in fresh media. These should be cultured for a further 24 h until the start of the assay. If the cells are too sparse, there may be too few for the assay.

### Preparation of staining stocks

**Timing: 20 min**

2. Prepare CellTrace Violet (CTV) stock solution, aliquot, and freeze for long-term storage.
  a. Allow the CTV stock tube (containing lyophilized CTV) and the provided anhydrous dimethyl sulfoxide (DMSO) solution to come to 20°C–25°C in the dark (approximately 10 min).
    **CRITICAL:** Anhydrous DMSO must be used when resuspending CTV. Water will cause CTV to deteriorate, even when frozen. The DMSO provided in the CTV kit is appropriate. Alternatively, another unopened anhydrous DMSO vial can be used. Do not use bench-top DMSO that is regularly used for freezing cells. The water contained within will reduce the shelf life of the CTV. Protect CTV stocks and stained cells from light.
  b. Briefly spin the CTV stock tube to collect the contents at the bottom.
  c. Open the CTV tube in a class II biological safety cabinet.
  d. Add 20 μL of anhydrous DMSO to the CTV stock tube.
    ***Note:*** This yields a 5 mM stock solution of CTV.
  e. Incubate upright at 20°C–25°C in the dark for approximately 10 min.
  f. Pipette up and down to mix.
  g. Briefly spin the tube to collect the contents at the bottom.
  h. Transfer 2 μL aliquots into sterile 0.5 mL reaction tubes.
    ***Note:*** A stock solution volume of 2 μL provides enough stain for 10 different target cells when stained at a 1:500 dilution (10 μM final concentration) in 100 μL of staining solution.
  i. Seal the tubes with parafilm and store the aliquots in airtight containers with desiccant packets at −20°C.
    ***Note:*** We regularly store CTV aliquots for two months at −20°C and have used them successfully to stain and detect target cells up to 72 h post-labeling.
    **CRITICAL:** Avoid multiple freeze-thaw cycles with CTV stocks. We regularly use CTV after a single freeze/thaw cycle and still achieve robust CTV signals. It is important that the containers are airtight to avoid water vapor entering the tubes.
3. Prepare CellEvent Caspase-3/7 Green solution and make aliquots.
  a. Allow the Caspase-3/7 Green stock tube containing 38 μg lyophilized reagent to come to 20°C–25°C in the dark (approximately 2 min).
  b. Briefly spin the Caspase-3/7 Green stock tube to collect the contents at the bottom.
  c. Open the Caspase-3/7 Green tube in a class II biological safety cabinet.
  d. Add 100 μL of sterile Dulbecco’s phosphate-buffered saline (DPBS, 20°C–25°C) without calcium or magnesium, yielding a 380 μg/mL stock solution.
  e. Incubate upright at 20°C–25°C in the dark for approximately 10 min.
  f. Pipette up and down to mix.
  g. Briefly spin the tube to collect the contents at the bottom.
  h. Transfer 10 μL aliquots into 0.5 mL reaction tubes.
    ***Note:*** A 10 μL stock volume provides enough stain for 100 wells when each well is stained with a 1:1,000 end dilution of Caspase-3/7 Green in 100 μL.
  i. Seal the tubes with parafilm and store the aliquots in airtight containers at −20°C.
    ***Note:*** We regularly store Caspase-3/7 Green aliquots for two months at −20°C and have successfully used them to stain apoptotic cells in cytotoxicity assays.
    **CRITICAL:** Avoid multiple freeze-thaw rounds with Caspase-3/7 Green stock solutions.

### Pilot assay and cytometer configuration

**Timing: 26 h (including a 24 h incubation for the pilot assay)**

The flow cytometer used to establish this method was a BD LSR Fortessa X-20 fitted with a High Throughput Sampler (HTS). It was equipped with ultraviolet (355 nm), violet (405 nm), blue (488 nm), yellow-green (561 nm), and red (637 nm) lasers. The complete list of detectors is in Table S1. However, the current protocol has been designed to be suitable for use with other instruments (Tables S2–S4), and comparable results were observed with different cytometers (Figure S1). As each flow cytometer is unique, the user should carry out a pilot assay and optimize the cytometer settings for each machine before undertaking experiments with precious target or effector cells.

Using the methods outlined in the “step-by-step method details” section, prepare a pilot assay and adjust the cytometer settings for optimal signal detection.

**Figure 1:**
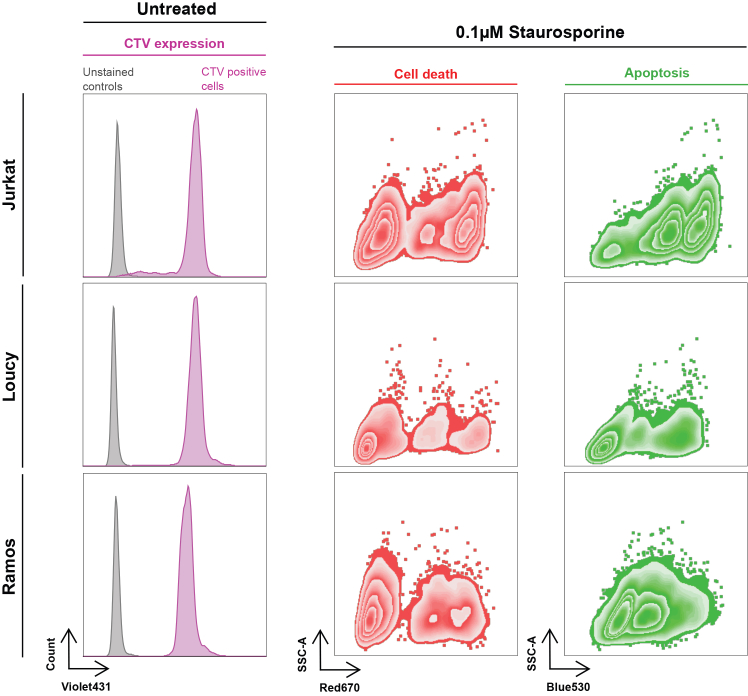
Detection of CTV, Cytotox Red, and Caspase-3/7 Green-positive events Jurkat, Loucy, and Ramos cells were stained with 100 μL DPBS containing 10 μM CTV for 20 min at 37°C or left unstained. Cells were washed and cultured for 24 h at 37°C in the presence of 0.1 μM staurosporine, or left untreated. Live and apoptotic cells were detected using Cytotox Red and Caspase-3/7 Green, respectively. Events shown were gated on Non beads/Cells/Single Cells SSC/Single Cells FSC.

4. Prepare a pilot assay and adjust the cytometer settings.
  a. Treat a range of unstained and CTV-stained cell lines with 0.1 μM staurosporine.
  b. Incubate cells for 24 h at 37°C with 5% CO_2_.
  c. Spike in untreated cells to act as the negative control population.
    ***Note:*** Staurosporine-treated cells serve as positive controls for cell death and apoptosis (Figure 1).
    ***Note:*** Different cell types may exhibit different fluorescence intensities. Accordingly, several different cell types should be used to determine the detector voltages. To ensure fluorescent signals from all cell types are on scale, voltage settings may be determined using cells with the brightest signals.
  d. For staurosporine-treated cells, prepare a range of non-stained, single-stained, and fully-stained samples using the methods outlined in the ‘Step-by-step method details’ section.
  e. Complete step 4d for staurosporine untreated cells.
  f. Prepare a sample with fluorescent counting beads diluted in DPBS.
  g. Without recording, analyze samples on a flow cytometer with all detectors enabled.
    ***Note:*** At least, the cytometer should be equipped with violet, blue, and red lasers.
  h. Adjust the cytometer voltages for forward scatter (FSC) light and side scatter (SSC) light.
    ***Note:*** The apparently healthy cell population should be easily resolved from dead and dying cells and debris while remaining on scale (Figure S2).
  i. Adjust the cytometer voltages for the fluorescence detectors.
    ***Note:*** Ensure to analyze forward and side scatter light area, height, and width signals on a linear scale and the fluorescent area signals on a log scale.
    ***Note:*** The majority of positive events from the full-treatment, single-stain controls should appear at approximately 10^4^ for each fluorescent detector and the brightest events should be no brighter than 10^5^.
    ***Note:*** Histograms instead of dot plots may be used here to aid in population visualization (Figure S2).
  j. Without recording, using cells from untreated, unstained samples, adjust the voltages until the majority of negative events lie between 0 and 10^2^ for all detectors.
  k. Ensure that the fluorescent counting beads are also on scale.
    ***Note:*** Although this method of voltage setting is not recommended for complex multicolor experiments, it is adequate for the current protocol.
  l. Without recording, check the applicability of the voltages with full-stained samples from various treatments of various cell lines.
    ***Note:*** All signals should be on scale and the positive and negative populations should be easily distinguishable.
    **CRITICAL:** Correct setting of cytometer voltages is essential for the accurate measurement of cells by flow cytometry. It allows for the resolution of dim events without losing bright events off scale.^7^ The vast topic of correct cytometer voltage setting is reviewed elsewhere.^7^^,^^8^
  m. Record a range of treated, untreated, unstained, single-stained, and fully stained samples from various different cell lines.
5. Identify the optimal fluorescent detectors for each stain.
  a. Export the median fluorescence intensity (MFI) values for the positive and negative populations for each stain, for every single-stained, staurosporine-treated sample, for every detector.
  b. Export the standard deviation of the fluorescence intensity values for the negative populations for each stain, for every single-stained, staurosporine-treated sample, for every detector.
  c. Determine which detector is best suited for detecting the respective stain (see Figure S3), using the formula for the stain index detailed below.
    $$Stain\;index=\frac{{MFI}_{pos}-{MFI}_{neg}}{{2\sigma }_{neg}}$$
    Where:
    *MFI*_*pos*_ = median fluorescence intensity of the positive population.
    *MFI*_*neg*_ = median fluorescence intensity of the negative population
    *σ*_*neg*_ = standard deviation of the fluorescence intensity of the negative population.
  d. Complete steps 5a to 5c for beads using unstained cells as the negative population.
    ***Note:*** Beads demonstrate robust stain indexes across many detectors, giving significant flexibility in detector selection (Figure S3). We typically identify beads using detectors suitable for measuring BUV395 (Indo1violet) and PE fluorescence (Figure S4).
    ***Note:*** The distinct side scatter light properties of counting beads and cells mean that bead signals typically do not interfere with cell signals. To further reduce the risk of interference, beads should be detected in a channel where other detection reagents exhibit little fluorescence. If necessary, non-fluorescent light scatter signals can be used alone or in combination with fluorescent signals to identify bead populations (Figure S4 A). However, it is important to note that not all FSC and SSC light parameters will allow for the differentiation of beads from cells (Figure S4 B).
  e. For subsequent experimental assays, use the detector with the greatest calculated stain index to detect each of the corresponding stains.
    ***Note:*** We found that CTV should be measured with a detector suitable for BV421/Pacific Blue detection, Caspase-3/7 Green should be measured with a detector suited for FITC/AF488/GFP detection, while Cytotox Red may be measured with a detector capable of measuring AF647/APC, although there is significant flexibility in the detector requirements for Cytotox Red (Figure S3).
    ***Optional:*** The current protocol focuses on the use of target cells that do not express transgenic reporters (Table S5). However, stains can be adjusted for use with GFP-positive target cells. In this case, Cytotox Red is substituted for Cytotox NIR, and Caspase-3/7 Green is substituted for Caspase-3/7 Red (Figure S5; Table S6). For the application of the GFP panel, due to Cytotox NIR signal spill-over, Caspase-3/7 Red must be excited by the yellow-green laser, not the red laser. Additionally, Caspase-3/7 Red should be measured with a detector suitable for PE-CF594 detection. The possibility of combining the current protocol with cells expressing fluorescent reporters other than GFP may be possible, although this has not yet been investigated.
    ***Note:*** Propidium iodide or 7-AAD can be substituted as cost-effective alternatives to Cytotox Red and may be analyzed using detectors suited for PE-CF594 or PE-Cy5 measurement. Propidium iodide is exceptionally bright, allowing for excellent discrimination of live cells from dead cells (Figure S6). Due to the similarity of the spectral properties of Caspase-3/7 Red, propidium iodide or 7-AAD cannot be substituted into the GFP-CTV panel.
    **CRITICAL:** According to the Globally Harmonized System of Classification and Labeling of Chemicals (GHS) and the National Institutes of Health (NIH), propidium iodide causes skin irritation, serious eye irritation, and may cause respiratory irritation. It is also suspected of causing genetic defects. Use with caution. Avoid contact with skin, ingestion, or inhalation. Wear appropriate personal protective equipment. Dispose of according to local safety regulations.
    **CRITICAL:** According to the GHS and NIH, 7-AAD may be harmful or fatal if ingested, inhaled or touched. It is acutely toxic, capable of causing skin irritation, suspected of causing cancer, and suspected of damaging fertility or unborn children. Use with extreme caution. Avoid contact with skin, ingestion, or breathing fumes. Wear appropriate personal protective equipment. Dispose of according to local safety regulations.

## Key resources table

**Table:** 

REAGENT or RESOURCE	SOURCE	IDENTIFIER
**Biological samples**		

Human peripheral blood mononuclear cells (PBMCs)	Healthy donor	N/A
Human NK, T, γδ T Cells	Healthy donor	N/A

**Chemicals, peptides, and recombinant proteins**		

2-Mercaptoethanol 50 mM (1,000 ×)	Thermo Fisher Scientific	31350010
7-AAD (52.5 μg/mL)	Miltenyi Biotec	130-111-568
CellEvent Caspase-3/7 Green	Thermo Fisher Scientific	C10432
CellEvent Caspase-3/7 Red	Thermo Fisher Scientific	C10430
CellTrace Violet Cell Proliferation Kit	Thermo Fisher Scientific	C34571
CS&T Beads	BD Biosciences	656504
Dulbecco’s Phosphate Buffered Saline (DPBS) without calcium or magnesium	Anprotec	AC-BS-0002
FACS Clean Solution	BD Biosciences	340345
FACSFlow Sheath Fluid	BD Biosciences	342003
FACSRinse Solution	BD Biosciences	340346
Fetal bovine serum, superior (Heat inactivated)	Merck	S0615-500ML
GlutaMAX Supplement 200 mM (100 ×)	Thermo Fisher Scientific	35050038
HEPES buffer 1 M (50 ×)	PAN Biotech	P05-01100
Human IL-15, research grade	Miltenyi Biotec	130-093-955
Human IL-2 Recombinant Protein, PeproTech	Thermo Fisher Scientific	200-02-100UG
Human IL-7, research grade	Miltenyi Biotec	130-095-367
Human Male AB Serum (Heat inactivated)	Access Cell Culture	535-100-HI
ImmunoCult Human CD3/CD28 T Cell Activator	STEMCELL Technologies	10971
Incucyte Cytotox NIR Dye	Sartorius	4846
Incucyte Cytotox Red Dye	Sartorius	4632
MEM Non-Essential Amino Acids Solution (100 ×)	Thermo Fisher Scientific	11140035
NK MACS Basal Medium with NK MACS Supplement, human	Miltenyi Biotec	130-114-429
Parafilm	Heathrow Scientific	HS234526B-1EA
Penicillin-streptomycin (100 ×) (10,000 U/mL penicillin, 10 mg/mL streptomycin)	PAN Biotech	P06-07100
Precision Count Beads	BioLegend	424902
Propidium iodide (500 μg/mL)	BioLegend	421301
RPMI 1640 with L-Glutamine	Anprotec	AC-LM-0058
Sodium pyruvate 100 mM (100 ×)	PAN Biotech	P04-43100
Staurosporine solution 1 mM	Merck	569396-100UG
Trypan blue	Thermo Fisher Scientific	15250061

**Experimental models: Cell lines**		

Jurkat	DSMZ	ACC 282
Loucy	DSMZ	ACC 394
NK92	DSMZ	ACC 488
Ramos	DSMZ	ACC 603
Specific Gene edited effector cells	Variable	N/A
Specific Gene edited target cells	Variable	N/A

**Software and algorithms**		

BD FACSDiva	BD	Version 9.0.1
BioRender	BioRender	N/A, https://www.biorender.com/
Excel	Microsoft	2016, https://www.microsoft.com/en-us/microsoft-365/excel-c
FlowAI	Gianni Monaco et al., 2016.	Version 2.3.2, https://www.flowjo.com/exchange/plugin/flowai
FlowJo	BD Biosciences	Version 10.10.0, https://flowjo.com/flowjo10/download
Prism	GraphPad	Version 10.5.0, https://www.graphpad.com/
R	R Core Team	Version 4.5.0, https://cran.r-project.org/bin/windows/base/
Rstudio	Posit Software	Version 2025.09.1+401, https://posit.co/download/rstudio-desktop/

**Other**		

Benchtop microcentrifuge	Eppendorf	Centrifuge 5420
Benchtop centrifuge	Thermo Fisher Scientific	Heraeus Megafuge 16R
Class II biological safety cabinet	The Baker Company	SterilGARD III Advance°
CO_2_ incubator for tissue culture	Thermo Fisher Scientific	Heracell 240i
BD FACSAria II Cell Sorter	BD Biosciences	N/A
BD FACSCanto II Flow Cytometer	BD Biosciences	338962
BD FACSCelesta Flow Cytometer	BD Biosciences	660344
Hemacytometer. Improved Neubauer (cell counting chamber)	Assistent	40442002
Inverted light microscope for tissue culture	Zeiss	Telaval 31
BD LSRFortessa™ X-20	BD Biosciences	657427
Pipette aid	Integra	Pipetboy 2

## Materials and equipment

**Table:** R10 medium

Component	Amount required	Final concentration
RPMI 1640 with L-Glutamine	500 ml	1 ×
Fetal bovine serum, superior (Heat inactivated)	50 ml	10%

Store at 4°C for up to four weeks.

**Table:** R10Extra medium^a^

Component	Amount required	Final concentration
RPMI 1640 with L-Glutamine	500 ml	1 ×
Fetal bovine serum, superior (Heat inactivated)	50 ml	10%
HEPES buffer 1M (50 ×)	10 ml	20 mM
GlutaMAX Supplement 200 mM (100 ×)	5 ml	2 mM^b^
Sodium pyruvate 100 mM (100 ×)	5 ml	1 mM
MEM Non-Essential Amino Acids Solution (100 ×)	5 ml	1 ×
2-Mercaptoethanol 50 mM (1,000 ×)	500 μl	50 μM

Store at 4°C for up to four weeks.

a We use R10Extra for our cytotoxicity assays. It is the media we regularly use for culturing fastidious cells (including primary T cells and γδ T cells). However, the end user may use any media of their choice that does not influence the viability of their target or effector cells.

b Plus 300 mg/L L-Glutamine included in the base RPMI media.

**CRITICAL:** According to the GHS and the NIH, 2-Mercaptoethanol is harmful or fatal if ingested, inhaled, or touched. It is corrosive, acutely toxic, very toxic to aquatic life, capable of causing allergic skin reactions, and suspected of damaging fertility or unborn children. Use with caution. Avoid contact with skin, ingestion, or breathing fumes. Wear appropriate personal protective equipment. Dispose of according to local safety regulations.

**Table:** NK medium

Component	Amount required	Final concentration
NK MACS Basal Medium, human	500 ml	1 ×
Human Male AB Serum (Heat inactivated)	25 ml	5%
NK MACs Supplement (100 ×)	5 ml	1%

Store at 4°C for up to four weeks.

## Step-by-step method details

### Target and effector cell preparation

**Timing: 1–2 h (depending on the number of different target and effector cells)**

**Figure 2:**
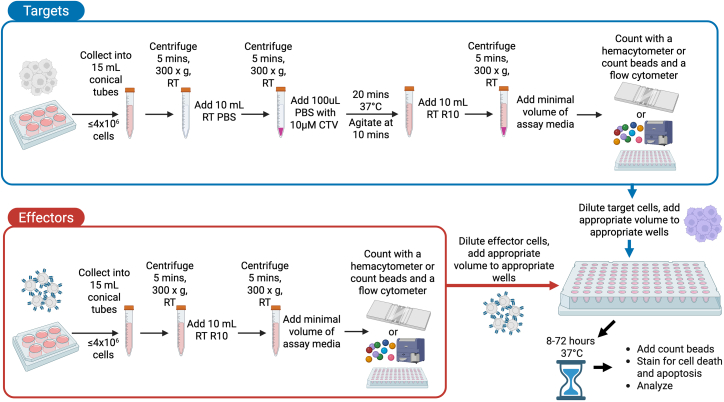
Overview of the preparation of cells for the cytotoxicity assay

This section includes detailed instructions for collecting, washing, staining, counting, plating, and incubating target and effector cells (Figure 2). For the preparation of cells for co-culture, conduct all cell manipulations in a class II biological safety cabinet with proper sterile technique.

To differentiate target cells from effector cells, at least one population must be labeled with CTV prior to co-incubation. CTV is designed to be used in live cell assays for tracking cell proliferation; it is non-toxic and does not cause a significant reduction in cell growth over 72 h (Figure S7). CTV signal intensity diminishes with cell divisions. However, we have successfully used it to track rapidly dividing Jurkat and Ramos cells for up to 72 h (Figure S8). An alternative to CTV is Tag-it Violet from BioLegend, although we have not investigated its use in the current protocol.

***Note:*** Even if target or effector cells are GFP-positive, CTV labeling of target cells must be completed. GFP fluorescence is often lost in dead cells (Figure S9). Without CTV to distinguish targets from effectors, the CTV-negative effector cell gate can be contaminated by dead target cells. Loss of GFP indicates a general reduction in cell viability of previously GFP-expressing cells. However, not all GFP-expressing target cells that lose their GFP signal are dead (Figure S10). Conversely, not all GFP-positive cells are viable. Furthermore, GFP-positive cells may be apoptotic, as indicated by caspase-3/7 activity. In contrast, cells do not lose their CTV fluorescence when dead or dying (Figure S11). Taken together, in the context of the current protocol, GFP fluorescence alone should not be relied upon as an identifier of viable non-apoptotic target cells.

***Note:*** If desired, both target and effector cells can be labeled. However, the fluorescent molecules used to identify cells must exhibit distinct spectral properties from each other, and also distinct spectral properties from those reagents used to quantify cell death and apoptosis. If target cells are labeled with CTV, effector cells may be labeled with CellTrace Blue, for example.

***Note:*** For the examples given in the current work, cytotoxicity assays were carried out in R10Extra. This includes assays with cells otherwise routinely cultured in alternative media. The authors have not investigated the application of different media for the current protocol. Also, co-culture cytotoxicity assays were carried out in in the presence of exogenous cytokines. However, the authors have performed co-culture cytotoxicity assays without cytokine supplementation for CAR-T effector cells and observed similar target cell death. If supernatants will be collected for cytokine analysis, cytokine supplementation is not recommended. Nevertheless, some fastidious cells may require the presence of exogenous cytokines, and end users are free to complete cytotoxicity assays with or without exogenous cytokine supplementation as required.

**CRITICAL:** Before undertaking large experiments, we strongly recommend users conduct small pilot cytotoxicity assays with target and effector cells that are less precious to allow for cytometer detector selection, voltage optimization, and protocol familiarity.

1. Preparation of target cells.
  a. Collect target cells into 15 mL sterile conical centrifuge tubes.
    ***Note:*** The volume of cells collected will vary depending on the number of cells required and their density. The number of cells required is influenced by the number of variables to be tested (effectors, time points, target-to-effector ratios, etc.). To ensure adequate cell numbers and to confirm cell viability, cells can be counted with trypan blue and a hemacytometer before harvesting.
  b. Centrifuge cells at 300 × *g* for 3-5 min at 20°C–25°C.
    ***Note:*** If working with low cell numbers, ensure to centrifuge for the entire 5 min to minimize losses.
    ***Optional:*** If the initial volume of harvested cells is 1 mL or less, cells can be collected into 15 mL sterile conical centrifuge tubes, directly topped up to 15 mL with DPBS (20°C–25°C), and centrifuged at 300 × *g* for 5 min at 20°C–25°C. Supernatants can then be discarded, cells resuspended by agitation, and stained as outlined, starting from step f below.
  c. Discard supernatants and resuspend cells by agitation.
  d. Top up cells with 10 mL DPBS (20°C–25°C) and centrifuge as above.
  e. Discard supernatants and resuspend cells by agitation.
  f. Thaw CTV stocks for 2 min at 20°C–25°C in the dark.
    ***Note:*** Frozen CTV stocks were prepared, aliquoted, and frozen previously (outlined in ‘Preparation of staining stocks’).
  g. Make a working stock solution by diluting the 5 mM CTV stock 1:500 in the appropriate volume of DPBS (20°C–25°C).
  h. Add 100 μL of 10 μM working stock CTV solution to each target cell to be stained.
    ***Note:*** This is enough to stain up to 4 × 10^6^ cells (Figure S12).
  i. Agitate to distribute the CTV evenly.
  j. Incubate cells for 10 min at 37°C in the presence of 5% CO_2_ in the dark.
  k. Agitate to redistribute the CTV evenly.
  l. Incubate for a further 10 min in the CO_2_ incubator.
    ***Note:*** Use the CTV working stock solution immediately. Do not store it for future use.
    ***Note:*** As demonstrated (Figure S12), 100 μL of 10 μM working stock of CTV is sufficient to stain 4 × 10^6^ Jurkat, Loucy, or Ramos cells. We have used this volume and concentration of CTV to successfully stain many other cell types, including primary cells. However, to ensure successful staining of desired target cells, the user may wish to titrate the CTV concentration or volume before performing a cytotoxicity assay.
    **CRITICAL:** Do not allow cells to incubate in the DPBS CTV mix for more than 20 min, as it contains DMSO and little protein. Prolonged exposure to these conditions may cause a substantial reduction in cell viability.
  m. Top cells up to 10 mL with R10 (20°C–25°C) and centrifuge as above.
  n. Discard the supernatant and resuspend the cells in a minimum volume of R10Extra media.
    ***Note:*** To avoid excessive dilution of cells, use a volume lower than the final volume required for the assay.
    ***Optional:*** At this stage, a small volume of CTV-stained cells can be analyzed on a flow cytometer. Compare this to an unstained control to ensure the CTV staining was successful before continuing.
  o. Collect 10 μL aliquots of cell samples into separate wells of a round-bottom 96-well plate.
  p. Add 80 μL R10 to each sample.
  q. Add 10 μL of Precision Count Beads.
    **CRITICAL:** Vortex the Precision Count Beads for 30 s before adding to the cell suspensions. If beads are not properly resuspended, the cell counts will not be accurate.
  r. Add propidium iodide to a final concentration of 0.5 μg/mL.
  s. Analyze using a flow cytometer equipped with a 96-well plate reader.
  t. Calculate the cell concentration using the equation:
    $$\frac{cells}{mL}=\left(\frac{c_{n}\times b_{v}}{b_{n}\times c_{v}\;}\right)\times b_{c}$$
    Where:
    c_n_ = total number of live single cell events recorded on the flow cytometer
    b_v_ = total volume of beads added to the counted sample (mL).
    b_n_ = total number of bead events recorded on the flow cytometer
    c_v_ = total volume of cells added to the counted sample (mL).
    b_c_ = stock concentration of beads (per mL) added to the counted sample
    ***Note:*** When preparing few different target cells, count them with a hemacytometer and trypan blue. When preparing many different target cells, count them with fluorescent counting beads and a flow cytometer as described above.
    ***Note:*** The dilution factor due to the R10 or propidium iodide does not need to be considered for this equation. The calculation is based on the ratio of cell events to bead events. This ratio remains the same even with a dilution due to added media. If the volumes of cells and beads added are known, and the concentration of beads is known, then the concentration of cells can be calculated.
    ***Note:*** Increasing the bead volume will increase the accuracy of each individual count but will also increase the cost. Similarly, increasing the cell volume used for counting will improve counting accuracy but reduce the cell volume available for downstream applications.
    ***Note:*** Although the gold standard for counting cells is a hemacytometer, it is cumbersome for high-throughput applications when many cell counts are required. Fluorescent counting beads in combination with a flow cytometer offer a robust alternative for cell counting when many samples must be counted (Table S7). Performing subsequent dilution calculations in an Excel table automates the calculation process and saves considerable time when handling many samples.
    **CRITICAL:** Achieving accurate cell counts is essential for the accuracy and success of a cytotoxicity assay.
  u. Resuspend cells in R10Extra to the desired concentration in the desired volume.
    ***Note:*** Cells should be diluted to a concentration such that the desired number of cells is contained in a final volume of 50 μL.
    ***Note:*** We typically add between 1,500 and 5,000 target cells and 1,500 to 5,000 effector cells per well. However, when targeting rare cell populations, low yields may limit the number of target cells available. We have also had success using as few as 500 target cells per well.
  v. Add 200 μL sterile water or DPBS to the edge wells.
    ***Note:*** These wells typically evaporate more rapidly than others, which may negatively affect cell growth, viability, and downstream calculations that rely on volume.
  w. Add 100 μL of target cells to the ‘no effector’ control wells of a 96-well round-bottom plate.
  x. Add 50 μL target cells to all wells that will receive both target and effector cells.
    ***Note:*** 96-well plates with round bottom wells are used in this assay. This allows for the use of fewer target and effector cells while still obtaining accurate results. One rate-limiting factor of cell cytotoxicity is the physical contact between target and effector cells.^9^^,^^10^ Round-bottom well plates ensure close contact between target and effector cells even when cell numbers are low.
    ***Optional:*** Volumes can be modified to change the number of target and effector cells in each well in order to adjust the target to effector ratios.
2. Prepare single stain controls for compensation.
  ***Note:*** Although this protocol is deliberately optimized to minimize spectral overlap between stains (Figures S13 and S14), it is best practice to apply compensation whenever multiple colors are used in flow cytometry. Due to changes in cytometer performance over time, it is advisable to record new compensation controls every day.
  ***Note:*** For high-quality compensation, single-stain controls should be at least as bright as your brightest sample. Therefore, the slowest-growing target cells labeled with CTV should be used for the CTV single-stain.
  ***Note:*** Healthy, untreated, unstained cells can be spiked into the single-stain controls to serve as the negative populations for compensation. This is preferred over a universal negative control because some stains can increase background fluorescence. This must be accounted for when compensating.
  ***Note:*** Fluorescent counting beads do not need to be compensated for.
  a. Add 100 μL of media containing between 30,000 and 90,000 of the slowest growing CTV labeled cells to an otherwise unoccupied well.
  b. Add 100 μL of media containing between 30,000 and 90,000 unlabeled cells of the same cell line to three further unoccupied wells.
    ***Note:*** One unstained well will be combined with the CTV-labeled cells prior to analyzing the plate. This will make CTV-positive and CTV-negative populations for the CTV-stained control.
  c. Add 0.1 μM staurosporine to two of the remaining unlabeled cell-containing wells.
    ***Note:*** One of these wells will be stained with Cytotox Red only, and the other will be stained with Caspase-3/7 Green only.
  d. Place the plate into the incubator until the effector cells have been prepared.
    ***Note:*** 0.1 μM staurosporine exposure for 24 h is effective at inducing apoptosis and cell death in several different cell lines. At this concentration after 24 h, a proportion of live, non-apoptotic cells is present that acts as the negative population for controls (Figure S15). If no cells survive the overnight treatment with staurosporine, unstained, untreated cells can be added to the staurosporine-treated single stain controls. The untreated cells will act as the negative control population for compensation. Add unstained, untreated cells after the 24 h incubation, and before adding Cytotox Red or Caspase-3/7 Green.
    ***Note:*** Some treatments may cause cells to autofluoresce. Analyze beads with a detector that distinguishes cells despite stains or treatment-induced autofluorescence. For example, Jurkat cells exhibit low autofluorescence in the UV379 detector when co-cultured with NK92 cells or left untreated (Figure S16). However, cells exhibited strong autofluorescence in this channel after treatment with 0.1 μM staurosporine. Despite this, the bead population was still discernible.
3. Preparation of effector cells
  a. Collect effector cells into 15 mL sterile conical centrifuge tubes.
    ***Note:*** The volume of cells collected will vary depending on the number of cells required and their density. The number of cells required is influenced by the number of different variables to be assessed (number of different target cells, time points, target to effector ratios, etc.).
  b. Centrifuge cells at 300 × *g* for 3–5 min at 20°C–25°C.
    ***Note:*** If working with low cell numbers, ensure to centrifuge the cells for the entire 5 min to minimize losses.
    ***Optional:*** If the initial volume of harvested cells is 1 mL or less, cells can be collected into 15 mL sterile conical centrifuge tubes, directly topped up to 15 mL with R10Extra (20°C–25°C), and centrifuged at 300 × *g* for 5 min at 20°C–25°C. Cells can then be prepared as outlined, starting from step f below.
  c. Centrifuge cells at 300 × *g* for 3–5 min at 20°C–25°C.
  d. Discard supernatants and resuspend cells by agitation.
  e. Top cells up to 10 mL with R10Extra (20°C–25°C) and centrifuge as above.
  f. Discard the supernatant and resuspend the cells in a minimum volume of R10Extra.
    ***Note:*** To avoid excessive dilution of cells, use a volume lower than the final volume required for the assay.
  g. Count cells using a hemacytometer and trypan blue, or with fluorescent counting beads and a flow cytometer as described for target cells in the “Preparation of target cells” section above.
  h. Resuspend cells in R10Extra to the desired concentration in the desired volume.
    ***Note:*** Cells should be diluted to a concentration such that the desired number of cells per well is contained in 50 μL.
  i. Add 100 μL of effector cells to the ‘no target’ control wells of the 96 round-bottom well plate.
    ***Note:*** These control wells indicate the background cell death in effector cells and allow estimation of effector cell death due to phenomena such as activation-induced cell death (AICD) or target cell cytotoxicity.
  j. Add 50 μL of effector cells to the appropriate wells.
    ***Optional:*** Same-day completion of cytotoxicity assays is critical for rapid assessment of novel effector cells. Target cell killing can be observed after 8 h using the current protocol (Figures S15 and S17). When conducting an 8 h cytotoxicity assay, cells must be centrifuged in 96-well round-bottom plates at 300 × *g* for 3 min before incubation. This allows for rapid contact between effector and target cells.
    ***Optional:*** Volumes can be modified to change the number of target and effector cells in each well to adjust the target to effector ratios.
    ***Optional:*** Staurosporine can be added to additional target cell only wells to serve as cell death and apoptosis positive controls. Furthermore, Jurkat cells are typically particularly sensitive to killing by WT NK92 or primary NK cells (Figure S18). These may also be included as positive controls.
  k. Incubate cells for the desired timeframe at 37°C, 5% CO_2_ in a humidified incubator.
    ***Note:*** Cytotoxicity has been detected in as few as 8 h with the current protocol. CTV is detectable even in rapidly dividing cells up to 72 h.

### Analysis of the cytotoxicity assay

**Timing: 90 min for a single plate, including staining**

This section includes instructions for endpoint staining of the cytotoxicity assay, data acquisition on a flow cytometer, and data analysis.

**Figure 3:**
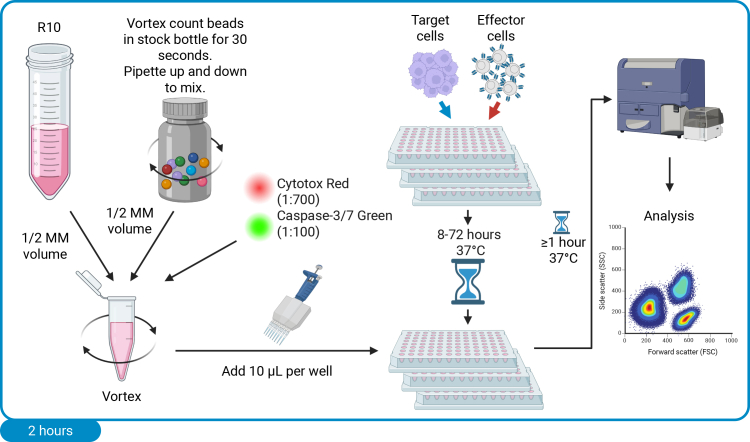
An overview of the ‘add-incubate-analyze’ no centrifugation end point staining protocol Cytotox Red, Caspase-3/7 Green, and Counting beads are non-toxic thus several plates can be stained in parallel and read sequentially. MM, master mix.

The endpoint process for this assay is quick and uncomplicated. It follows an ‘add-incubate-analyze’ format with no centrifugation or wash steps (Figure 3). This significantly reduces technical complexity and enables the processing of several plates simultaneously, increasing the assay’s high-throughput capacity.

***Note:*** Cytometers should be switched on, and the optical system temperature allowed to stabilize for at least 30 min before recording any quality control or experimental samples. For optimal cytometer performance, quality control (Cytometer Setup and Tracking (CS&T) for Becton Dickinson (BD) machines) should be performed on each machine every day before use. This is completed using CS&T beads and an automated process on FACSDiva software.

**Figure 4:**
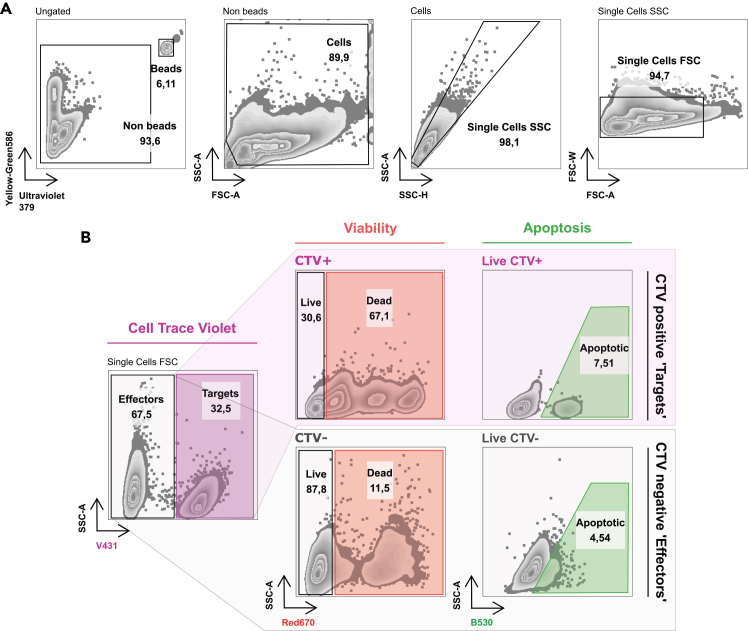
Cytotoxicity assay gating strategy Loucy cells were stained with 100 μL DPBS containing 10 μM CTV for 20 min at 37°C. Cells were washed and co-cultured with WT NK92 cells (Target:effector ratio of 1:1) for 24 h at 37 °C. To detect cell death and apoptosis, cells were incubated with a 1:7,000 dilution of Cytotox Red and a 1:1,000 dilution of Caspase-3/7 Green for 1 h at 37°C in the dark. To quantify cell numbers, 10 μL of fluorescent counting beads (1 × 10^6^ beads/mL) was added to each well. Beads and single cells (A), and target cells, dead cells, and apoptotic cells (B) were identified with the depicted gating strategy.

4. End point staining
  a. Calculate the volume of master mix required in microliters for the endpoint master mix using the formula:
    mastermixvolume(μL)=w×v×m
    where:
    w = the number of wells in the assay that require master mix.
    v = the volume of master mix required for each well in microliters.
    m = the multiplication factor required to ensure enough master mix is present despite volume losses due to pipette error.
    ***Note:*** A multiplication factor of 1.1 to 1.2 is advised to ensure enough volume is present without wasting valuable reagents.
    ***Note:*** Wells for single stain controls do not need to be taken into account for the master mix. They must be stained separately, not with the endpoint master mix. Beads should not be included with the single stain controls.
  b. Add half of the volume calculated above (step 4a) of R10 (20°C–25°C) to a 1.5 mL reaction tube.
  c. Vigorously vortex the precision count beads in their stock bottle for 30 seconds.
  d. Pipette them up and down two to three times with a 1 mL pipette to mix.
  e. Add the same volume of precision count beads to the endpoint master mix as was added for R10 in step 4b.
    ***Note:*** This will bring the total volume up to the calculated volume in step 4a.
    ***Note:*** Fluorescent counting beads are added to the wells of the samples to be counted. These act as a standard with which the exact volume of a recorded sample can be determined. This is necessary for cell number quantification.
  f. Thaw Caspase-3/7 Green and Cytotox Red for 5 min in the dark at 20°C–25°C.
  g. Add a 1:100 dilution of Caspase-3/7 Green to the endpoint master mix.
  h. Add a 1:700 dilution of Cytotox Red to the endpoint master mix.
    ***Optional:*** Here, the protocol may be adjusted to conduct multiple time points or a rechallenge assay. Before adding the endpoint master mix, transfer a known volume of cells to a new 96-well round-bottom plate, add the master mix, and perform the endpoint analysis in this plate. If the initial time point is less than 72 h, wells in culture can be topped up with media, and the remaining cells cultured until the next time point. However, this will significantly reduce the number of cells for analysis for each time point. Alternatively, regardless of time point, effector cells can be challenged a second time with fresh target cells labeled with a different fluorescent marker (such as CellTrace Blue). This forms a rechallenge assay.
  i. Vortex the endpoint master mix, then add 10 μL to each well using a multichannel pipette.
  j. Pipette wells up and down to mix and incubate the plate for 1 h at 37°C, 5% CO_2_ in the dark.
    ***Note:*** Cytotox Red and Near Infrared (NIR), and CellEvent Caspase-3/7 Green and Red are manufactured for live cell imaging. They are inherently non-toxic and label dead and apoptotic cells over extended incubation times.
    ***Note:*** We have determined the compatibility of Cytotox Red and Caspase-3/7 Green by staining cells in a cytotoxicity assay using either stain alone or both stains in combination. Similar frequencies of dead or apoptotic cells were observed regardless of multiplexing (Figure S19).
5. Record data on a flow cytometer
  a. Ensure instrument quality control was carried out.
    ***Note:*** This is analogous to CS&T on BD cytometers.
    **CRITICAL:** Instrument quality control should be carried out every day before analyzing experimental samples.
  b. Configure the cytometer using the voltage settings determined in the ‘Pilot assay and cytometer configuration’ section.
  c. Set up the gating strategy and plots similar to those depicted in Figure 4.
  d. Use appropriate fluorescent detectors to discriminate beads from cells (Figure 4A).
  e. Use FSC and SSC parameters to identify single cells (Figure 4A).
  f. Detect target and effector cells, live cells, and apoptotic cells using detectors capable of detecting BV421, APC, and GFP, respectively (Figure 4B).
    **CRITICAL:** Before recording controls or samples, analyze a range of different stained and unstained, treated and untreated test samples in tube format to ensure that signals are consistent with the pilot assay and that events are on scale.
  g. Adjust cytometer settings if necessary and reanalyze test samples.
  h. Once cytometer settings are appropriate, record single stain compensation controls and apply compensation to the experiment.
    ***Note:*** If desired, instead of applying compensation now, single-stained controls can be recorded at this point and then compensation retrospectively applied during analysis.
  i. Attach and initialize the HTS plate loader.
  j. Record experimental samples in plates on the flow cytometer using the HTS plate loader.
    ***Note:*** An example of the HTS settings regularly used while recording data using the current protocol on a BD LSRFortessa X-20 can be found in Table S8. Analyzing a 96-well plate containing 60 wells using the HTS with these settings takes approximately 25 min.
    ***Optional:*** If the cytometer is not equipped with a plate loader, the wells can be transferred to 5 mL FACS tubes and run on a regular Sample Injection Port (SIP). This will drastically reduce the high-throughput nature of the current protocol.
    **CRITICAL:** Ensure all voltages are set correctly before recording any samples. If voltages are set too high, positive events may be off scale. Events that are off-scale cannot be resolved from each other (Figure S2). If off-scale events are present, voltages should be adjusted to ensure events are on scale, and all samples should be recorded again. If samples cannot be recorded again, it is best practice to exclude off-scale events from subsequent analysis. If voltages are set too low, positive populations are difficult to distinguish from negative populations. Adjusting cytometer voltages for the current protocol is discussed in the ‘Pilot assay and cytometer configuration’ section. Once fluorescent parameter voltages have been set, they should remain the same. This ensures comparability between samples. To account for variations in size and granularity of target or effector cells, FSC and SSC voltages may be adjusted for different cell types.
    **CRITICAL:** When recording using a plate loader, ensure the sample volume is set low enough that not all the sample in each well is collected. If excess sample is collected, air may enter the flow system. This can render current and future samples unusable. The flow cell and fluidics lines may need to be primed and degassed before continuing. If identified too late, the results from an entire plate may be unusable. If critical wells have been rendered unusable, top them up to 200 μL with R10 and re-record the appropriate wells. The cell concentration will be greatly reduced, but these critical wells may be salvaged.

## Expected outcomes

The successful completion of this protocol results in the accurate quantification of cell death, apoptosis, and the absolute cell number of both target and effector cells. This high-throughput cytotoxicity assay can be adapted for use with many different target or effector cell types that are cultured in suspension, and it is suitable for flow cytometers with a range of fluorescent detection capabilities. Target cells are easily distinguishable from unstained effector cells by their CTV expression for up to 72 h after cytotoxicity assay initiation. Cytotox Red allows for the identification of dead cells, while Caspase-3/7 detects cells undergoing apoptosis. Fluorescent counting beads allow for the quantification of absolute cell numbers.

**Figure 5:**
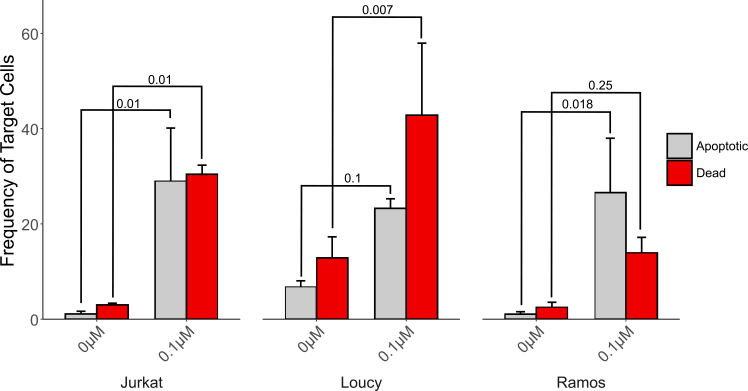
Induction of cell death and apoptosis by staurosporine Jurkat, Loucy, and Ramos cells were treated with 0.1 μM staurosporine for 24 h or left untreated. Dead cells were detected using Cytotox Red, and apoptotic cells were detected using Caspase-3/7 Green. Adjusted p-values are shown. Two-way ANOVA with Tukey’s multiple comparisons. n = 3. Data are represented as mean + SEM.

Using the current protocol, cell death and apoptosis in Jurkat, Loucy, and Ramos cells were measured in response to staurosporine (Figure 5). Staurosporine caused a time- and dose-dependent increase in cell death and apoptosis (Figure S15).

**Figure 6:**
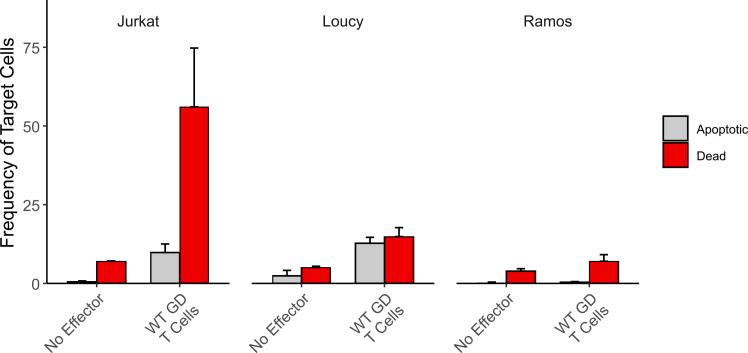
Induction of cell death and apoptosis by primary γδ T cells Jurkat, Loucy, or Ramos cells were stained with 100 μL DPBS containing 10 μM CTV for 20 min at 37°C. After washing, target and effector cells at a 1:1 ratio were added to the appropriate wells of a 96-well round-bottom plate. WT γδ T cells were expanded for 12-14 days with IL-2, IL-4, and Concanavalin A. γδ T cells were co-cultured with CTV-positive targets for 24 h at 37°C. Dead cells were detected using Cytotox Red, and apoptotic cells were detected using Caspase-3/7 Green. n = 2–4. Data are represented as mean + SEM. GD, γδ.

Jurkat cell death and apoptosis were increased in response to primary or immortalized NK cells, regardless of CAR expression (Figure S18). This reflects the high sensitivity of Jurkat cells to innate killing by natural killer cells,^11^^,^^12^ and may serve as a positive control for end users. Using the cytotoxicity assay described here, it was observed that γδ T cells caused an increase in Jurkat and Loucy cell death and apoptosis (Figure 6).

**Figure 7:**
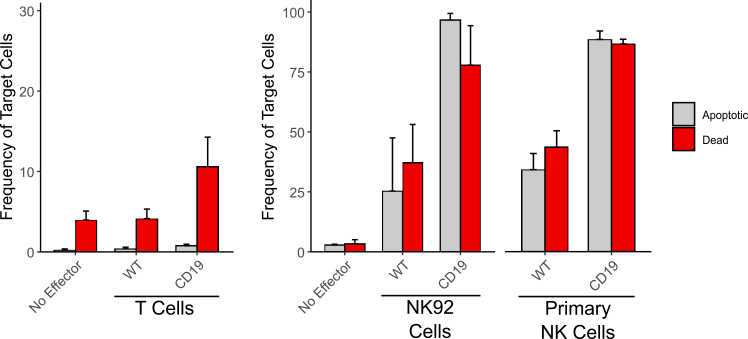
Induction of Ramos cell death and apoptosis by primary CAR-T, CAR-NK92, and CAR-NK cells CD19-positive Ramos cells were stained with 100 μL DPBS containing 10 μM CTV for 20 min at 37°C. After washing, target and effector cells at a 1:1 ratio were added to the appropriate wells of a 96-well round-bottom plate. Cells were co-cultured for 24 h at 37°C. Dead cells were detected using Cytotox Red, and apoptotic cells were detected using Caspase-3/7 Green. n = 2–4. Data are represented as mean + SEM.

Using the protocol described here, it was demonstrated that the frequency of dead Ramos cells increased in response to anti-CD19 CAR-T cells (Figure 7). Furthermore, the frequencies of dead Ramos cells and apoptotic Ramos cells were increased with WT NK92 cells, or WT NK cells, and drastically increased with NK92 or NK cells expressing an anti-CD19 CAR. Importantly, although only 75% of Ramos cells were killed by anti-CD19 CAR-NK92 cells, almost 100% of the remaining live cells were apoptotic. Similarly, anti-CD19 CAR-NK cells killed 85% of Ramos cells during the assay, and of the 15% that remained, almost 90% were caspase-3/7-positive.

**Figure 8:**
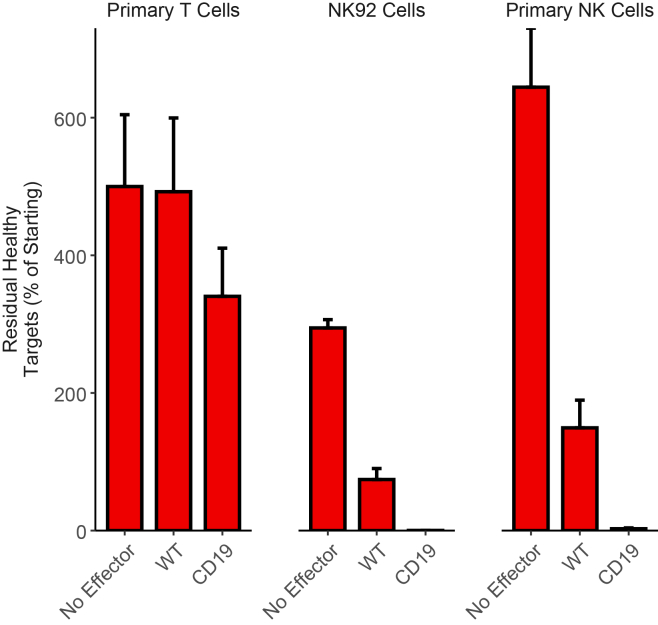
Absolute quantification of remaining viable, non-apoptotic Ramos cells CD19-positive Ramos cells were stained with 100 μL DPBS containing 10 μM CTV for 20 min at 37°C. After washing, target and effector cells at a 1:1 ratio were added to the appropriate wells of a 96-well round-bottom plate. Cells were co-cultured for 24 h at 37°C. Dead cells were detected using Cytotox Red, and apoptotic cells were detected using Caspase-3/7 Green. Remaining viable, non-apoptotic Ramos cells were quantified using counting beads, and the frequency of their starting cell number is presented. n = 2–4. Data are represented as mean + SEM.

The number of remaining viable, non-apoptotic Ramos cells was also quantified. This was completed using fluorescent counting beads. The number of remaining healthy cells was presented as a frequency of the starting viable cell number. WT NK92 and WT NK cells caused an increase in the frequency of dead and caspase-positive Ramos cells (Figure 7). However, a large proportion of viable Ramos cells remained (Figure 8). In contrast, live, non-apoptotic Ramos cells were almost undetectable when co-cultured with anti-CD19 CAR-NK92 or CAR-NK cells. This highlights the importance of determining the absolute numbers of remaining viable, non-apoptotic targets.

**Figure 9:**
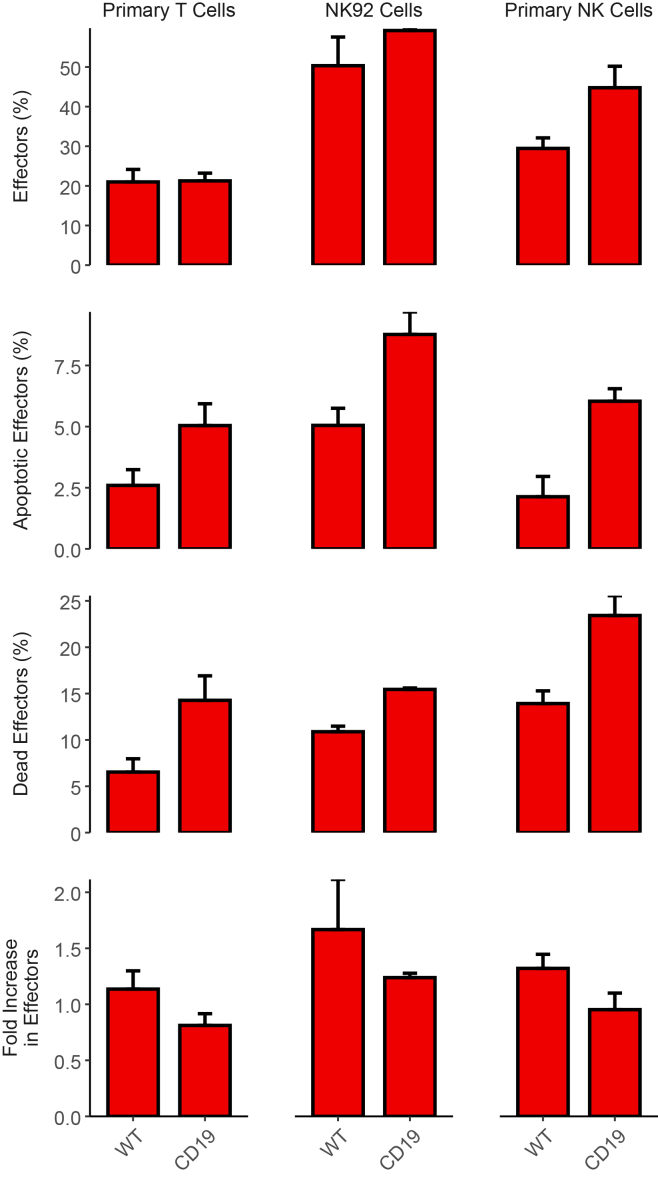
Quantification of co-culture impact on effector cells CD19-positive Ramos cells were stained with 100 μL DPBS containing 10 μM CTV for 20 min at 37°C. After washing, target and effector cells at a 1:1 ratio were added to the appropriate wells of a 96-well round-bottom plate. Effector cells were co-cultured with CTV-positive target cells for 24 h at 37°C. Dead cells were detected using Cytotox Red, and apoptotic cells were detected using Caspase-3/7 Green. Remaining viable, non-apoptotic effector cells were quantified using counting beads, and the frequency of their starting cell number is presented. n = 2–5. Data are represented as mean + SEM.

The impact of Ramos co-culture on effector cells was also quantified (Figure 9). At the conclusion of the cytotoxicity assay, higher frequencies of caspase-3/7-positive or dead anti-CD19 CAR-expressing effector cells were observed compared to WT controls. This indicates a potential loss of effector cell fitness and may reveal the requirement for effector cell optimization. The assessment of effector cell death and apoptosis is also crucial in the context of cytotoxic target cells, where reciprocal killing of effector cells by target cells may occur. Taken together, the impact of target-effector cell co-culture should be measured in both effector and target cells.

By combining an indicator of cell viability with an indicator of caspase-3/7 activity, users can quantify the frequency of dead cells and the frequency of those cells that are destined to die by apoptosis, yet have intact cell membranes. The use of counting beads enables quantification of the absolute number of healthy, live, non-apoptotic target cells, rather than relying on frequencies. This is important because frequencies of dead or apoptotic cells alone do not inform the user about the absolute number of residual healthy target cells in the co-culture, and target cell expansion is possible despite increased target cell death. Counting beads also allow users to understand the capacity for effector cell proliferation during co-culture with target cells. Cell membrane integrity loss, the induction of apoptosis, and the physical removal of identifiable target cells may occur at different time points during co-culture. By incorporating these different readouts into a single assay, users can gather information on each of these temporally distinct parameters and make informed conclusions on the cytotoxic potential of novel effector cells.

## Quantification and statistical analysis

Flow cytometry data is recorded using FACSDiva. However, due to its somewhat inflexible nature, we suggest using FlowJo for more detailed data analysis. Export FCS 3.0 files from FACSDiva and import them into FlowJo.

Before analysis, data should undergo quality control, and poor-quality data should be removed. FlowJo has an integrated process for inspecting flow cytometry data quality. However, users typically manually set time gates for each sample individually. This process is time-consuming, cumbersome, and prone to user bias. One efficient, unbiased method is to use the FlowJo plugin, FlowAI.^13^ This plugin automatically detects data that has no serious anomalies and allows users to select and analyze it. The so-called ‘GoodEvents’ can be exported for further analysis. The plugin also provides a detailed summary of the data removed. The automated nature of the plugin saves time and prevents user bias. For instructions on how to install and use FlowAI, see: https://docs.flowjo.com/flowjo/plugins-2/plugin-demonstration-videos/flowai/.

***Note:*** The statistical software environment ‘R’ is required for the use of FlowAI in FlowJo.

After quality control, data can be imported back into FlowJo for analysis. The gating strategy outlined in Figure 4 can now be used to analyze the results of the cytotoxicity experiment.

***Note:*** For data accuracy, only single cells should be included in downstream analysis.^7^^,^^14^ Several methods can be used for doublet discrimination during the analysis of flow cytometry data.^8^ It is common to use linearly acquired forward-scatter or side-scatter light signals to identify and remove doublet cells. This can be achieved by plotting the pulse area against the pulse height or width.^8^ However, some doublets may be overlooked in the first exclusion gate. Therefore, data may be passed through successive doublet gates.^7^^,^^15^ Also, different methods of doublet discrimination may be better suited to data from different flow cytometers. We advise users to include at least two doublet discrimination gates when analyzing flow cytometry data.

***Note:*** Different cells exhibit different FSC and SSC properties,^16^ have different autofluorescence properties,^17^ and will react to stains and treatments differently. Therefore, each individual cell line will require slightly different gate placements. During analysis, we suggest adjusting the gating strategy as needed for each different target cell type, but strictly applying the same gates when comparing samples with the same target cells.

## Limitations

The protocol detailed here is a robust method for simultaneously assessing both target and effector cell viability, apoptosis, and cell number. However, similar to all cytotoxicity assays in general, there are limitations.

This protocol is currently only applicable to target and effector cells grown in suspension culture. TrypLE or Accutase application at assay conclusion may release adherent target cells without affecting their viability, enabling analysis by flow cytometry. However, we have not yet investigated this. Alternatively, a live cell imager may be used to assess cytotoxicity in adherent cells. Regardless, in the context of suspension-grown target and effector cells, the current protocol remains a powerful tool to analyze target and effector cell outcomes.

It is challenging to distinguish between reversible changes in certain cellular processes and the permanent loss of cellular viability.^18^ Although there are many methods for assessing cell viability,^19^ cell death is measured in the current protocol by the detection of cell membrane permeability. This remains the gold standard for identifying dead cells.^20^ However, cell membrane integrity may be compromised in cells for reasons other than cell death.^21^^,^^22^ Also, cells may survive seemingly lethal membrane-permeability events, and many membrane repair mechanisms are known.^23^ Alternatively, cells destined to die may not yet have a compromised membrane, such as seen in early apoptosis. This could lead to false negatives in the current protocol and confirms the requirement for multiple indicators of cell health or viability (e.g., apoptosis indicators and absolute target cell counts employed in the current protocol). Regardless, cell membrane integrity remains one of the best indicators of cell viability.^18^^,^^19^

Apoptosis is measured in this protocol by the activation of caspases 3 and 7. However, caspase-3 activation has been documented in non-apoptotic cellular processes such as T cell activation^24^ and cellular differentiation.^25^ Due to the high-throughput nature of this protocol, it is impractical (and potentially inaccurate due to cell loss through centrifugation), and costly to stain every reaction for annexin-V binding.^26^ To assess the validity of caspase-detected apoptosis, annexin V binding could be carried out in parallel with caspase activation as a confirmatory assay. Regardless, caspase-3/7 activation remains a reliable and commonly used indicator of apoptosis.^26^

During the current protocol, cells are measured at the end of the assay and cannot be returned to culture. Therefore, no continuous time point analyses are available. To overcome this, the user may serially sample a single plate or may use multiple plates for multiple time points.

During the current protocol, cells are measured using a flow cytometer. Therefore, a visual representation of the cytotoxicity assay is unavailable. To overcome this limitation, a live cell imager may be employed. However, the measurement of cytotoxicity assays by flow cytometry is superior to live cell imaging in several (but not all) aspects. First, flow cytometry allows for the use of low cell numbers, which is critical when working with rare target or effector cells. Additionally, a flow cytometer can measure tens of thousands of cells in a homogenized suspension per second, sampling an averaged representation of the cell populations present. In contrast, live cell imaging relies on a relatively small area of focus which may be affected by uneven staining or cell distribution. Furthermore, due to limited plate compartments, many more plates, and therefore conditions, can be screened in a single day with flow cytometry-based methods. In addition, the high sensitivity of PMT detectors in a flow cytometer enables the use of substantially diluted reagents, drastically reducing costs. In general, cytometers typically offer more detector options than live cell imagers, providing greater flexibility. Therefore, new stains may be introduced without removing existing stains. Also, exported FCS files are relatively small compared to data-intensive microscopy images. This increases analysis speed and decreases data processing requirements. Lastly, if required, surviving target or effector cells may be sorted and used in downstream applications, rather than discarded.

These are, of course, generalizations. Depending on flow cytometer configuration, performance, or maintenance status, certain cytometers may be less sensitive or slower than newer live cell imagers. If visual confirmation is essential, a live cell imager can be employed to confirm a sub-sample of screened conditions or in parallel.

## Troubleshooting

### Problem 1

No or few events, very low fluorescence, very high fluorescence, unusual fluorescence signal, or low FSC or SSC (during test sample analysis using a tube, before starting to record a plate).

### Potential solution

• No cells in the sample. Transfer a sample to a tissue culture plate and check if cells are present with a microscope.
• Voltages of detectors are incorrectly set. Adjust detector voltages as outlined in the ‘Pilot assay and cytometer configuration’ section.
• Blocked lines, dirty flow cell, or air contamination of the flow cell. Acquire a test sample or beads. If unsuccessful, prime the flow cell. Acquire another test sample. If unsuccessful, allow FACS Clean to run for 3 min on HIGH. Allow FACS Rinse to run for 3 min on flow rate setting HIGH. Allow filtered deionized (DI) water to run for 3 min on HIGH. Check the FSC SSC and fluorescent properties of cells fresh from culture or from a non-valuable test sample. If the signal looks acceptable, attach the HTS coupler, reinitialize the HTS loader, and prime it. Record a test well from the plate. If unsuccessful, continue cleaning the cytometer with FACS Clean, FACS Rinse, and filtered DI water, and priming until signals return to normal. If this does not work, ask a flow cytometer technician to remove the blockage manually (using a wire-cleaning stylus). If signals do not return to normal, you may need to contact the responsible technician.
• Incorrect threshold. Adjust the threshold to allow smaller or larger particles to be registered as events. Ask the cytometer technician for assistance.
• The cytometer sheath fluid is empty. Ensure the containers are full, then prime the machine.
• The lasers are turned off. Ensure they are turned on.
• The machine is not functional. Conduct CS&T to check. Contact the technician if the cytometer is not performing accurately.

### Problem 2

Plate fails mid-run, and the data from some or all wells is unusable.

### Potential solution

• Blocked lines, dirty flow cell, or air contamination of the flow cell. Stop the plate. Collect the remaining volume of each well that must be reacquired and add to a new plate. Top these wells up to 200 μL with R10 and keep them in the dark until the problem is resolved. Prime and reinitialize the plate reader. The following applies to BD machines. If working with a cytometer from a different manufacturer, contact the responsible person. Try a test well with beads or a control sample. If this is unsuccessful, detach the HTS loader and prime the SIP twice. Try a test sample in a 5 mL tube. If this is unsuccessful, allow FACS Clean to run for 3 min on HIGH. Allow FACS Rinse to run for 3 min on HIGH. Allow filtered DI water to run for 3 min on HIGH. Try a test sample. If the signal looks normal, reattach the HTS coupler, reinitialize, and prime it. Run a test well. If unsuccessful, continue cleaning the cytometer with FACS Clean, FACS Rinse, and filtered DI water, and priming until signals return to normal. Once a test well looks normal, start recording the new plate with a larger recording sample volume. The cell concentration will be greatly reduced because there are now fewer cells in a larger volume. However, since we must always leave dead volume at the bottom of the well, and this is a fixed volume regardless of sample volume, the greater sample volume will mean a greater fraction of the well contents is recorded (compared to if we only topped up to 100 μL). Running a larger sample volume will take longer. For absolute cell counts, we do not need to account for this extra dilution when calculating cell numbers, as the calculation relies on the ratio of cells to beads. Since both cells and beads were diluted equally, the ratio remains the same.
• The cytometer sheath fluid or filtered DI water for the plate loader is empty. Ensure the containers are full, then prime the machine.

### Problem 3

The event rate is far higher than expected.

### Potential solution

• Cell number is too high. Ensure accuracy of counting when seeding cells for the cytotoxicity assay. See ‘Preparation of target cells’.
• Reduce cell seeding density.
• Record samples with a lower flow rate.
• Bacterial contamination. Ensure proper sterile technique and prepare cells in a class II biological safety cabinet. See ‘Preparation of target cells’. Check if the well is overly acidic or cloudy. Check if bacteria can be seen in the wells with a microscope. Test reagents for sterility and use new media if required. Add penicillin and streptomycin to the cytotoxicity assay if required. Avoid using water baths to warm media. They are a common source of bacterial contamination. Allow the media to warm to 20°C–25°C on a lab bench.
• Dirty flow cell or air contamination. Stop the plate. Troubleshot the problem as outlined for blocked lines, dirty flow cell, or air contamination as described for problem 1.

### Problem 4

Could not detect CTV positive cells.

### Potential solution

• Check the CTV positivity of a small sample of target cells before adding effector cells to the well during the assay setup. See ‘Preparation of target cells’.
• Target cells were killed with such efficiency that no intact CTV-positive cells are present. This is not something we have encountered, but in theory, it could happen. Reduce the number of effector cells. See ‘Preparation of effector cells’. Reduce the time frame of the cytotoxicity assay.
• Too many cells were stained at once, resulting in poor CTV-labeling efficiency. Ensure to stain a maximum of 4 × 10^6^ cells (‘Preparation of target cells’). Some target cells may require more stain than others. The staining volume may be increased to 200 μL, or the CTV concentration may be increased.
• Protein present during staining. To remove most of the protein from the media, ensure cells are adequately washed with PBS before staining (‘Preparation of target cells’).
• CTV concentration was too low. Check the concentration used. Try a range of concentrations.
• CTV stain is no longer functional. Prepare fresh aliquots. See ‘Preparation of staining stocks’. Substitute CTV for CFSE without using Caspase-3/7 Green during endpoint staining.
• Too few CTV-positive cells were added to the well. Adjust the target-to-effector cell ratio or carry out a cytotoxicity assay with various target-to-effector ratios. Ensure the accuracy of target and effector cell counts before the co-culture. See ‘Preparation of target cells’ and ‘Preparation of effector cells’.
• Assay co-culture was too long. Test a range of incubation lengths. We have found that, depending on the cell line, the CTV signal becomes very low after 72 h.
• The CTV detector voltages are set too low. Check machine voltages as outlined in the solutions to problem 1 and the ‘Pilot assay and cytometer configuration’ section.
• Incorrect gate setting on the SSC FSC plot. Ensure to include both the apparently healthy cells and the apparently dead cells in the SSC FSC gate. Do not include debris.
• An incorrect detector was used. Analyze with all detectors to determine the optimal detector. See ‘Pilot assay and cytometer configuration’ and Figure S3.
• Overcompensation. Try analyzing without compensation applied. Complete compensation again with new compensation controls if necessary. See ‘Prepare single stain controls for compensation’.
• Conduct CS&T to establish if violet detectors are functional. Contact the responsible technician if the cytometer is not performing accurately.

### Problem 5

High target cell or effector cell death or apoptosis even in target-only or effector-only controls.

### Potential solution

• Variation in background cell death and apoptosis is expected. However, increased cell death or apoptosis may be observed in cells that were unhealthy before the assay began. Ensure the cells are not overcrowded and that the media is not depleted for the 24 h leading up to the beginning of the assay. If required, cell viability can be assessed before the assay begins using trypan blue exclusion. See ‘Generation of target and effector cells’.
• For CTV-stained cells, ensure cells are not incubated in CTV for too long (‘Preparation of target cells’). Prolonged exposure to DMSO or conditions with very low protein concentrations can be detrimental to cell health. Users may stain cells with CTV in the presence of variable concentrations of Bovine Serum Albumin (BSA) to determine if cells can be adequately stained even in the presence of protein. This may improve fastidious cell viability. Investigate different incubation times for CTV staining to establish if a high frequency of CTV-positive cells may be achieved with shorter incubations.
• Cells were too dense by the end of the assay. Reduce the starting cell numbers in each well.
• Cell sparsity contributed to poor viability. Increase cell numbers at the start of the assay.
• Check the compatibility of the cells with the media used for the cytotoxicity assay.
• Check concentrations of stains and media additions. Reduce Cytotox Red and Caspase-3/7 Green staining concentrations.
• Add essential cytokines for fastidious cells (IL-2 for NK92 and T cells, etc.).
• Bacterial contamination. Check for the solution in the ‘potential solution’ to problem 3.
• The voltage settings for the dead cell detector may be too high. Run cells directly from culture without staining to assess positivity. Reduce the voltages so that the unstained control population is between 0 and 10^2^. Then adjust the detector sensitivity using a single-stain full treatment control. See ‘Pilot assay and cytometer configuration’.
• Incorrect gate setting on the SSC FSC plot. Ensure to include the apparently healthy cells and the apparently dead cells in the SSC FSC gate. Do not include the debris. See Figure 4A.
• Ensure cytometer lines are free of cleaning reagents.
• Conduct CS&T to establish if the detectors are functional. Contact the responsible technician if the cytometer is not performing accurately.

### Problem 6

High target cell death or apoptosis when incubated with negative-control effector cells, which should have little or no cytotoxicity.

### Potential solution

• Too many effector cells were added to the well. Adjust the target-to-effector cell ratio, or carry out a cytotoxicity assay with various target-to-effector cell ratios. Ensure the accuracy of target and effector cell counts before the co-culture. See ‘Preparation of target cells’ and ‘Preparation of effector cells’.
• Effector cells are strongly or non-specifically activated. Reduce the concentration of cytokines in the media used by effector cells.
• Certain target cells may be exceptionally sensitive to killing by certain effector cells (Figure S18). Test with a range of target cells.
• Effector cells are genuinely cytotoxic. Use a different negative control.
• The time frame was too long. Incubation with effector cells, even if they should exhibit low cytotoxic potential (wild-type cells, antigen specificity controls, or truncation controls in the case of CAR-T cells, for example), may eventually lead to nonspecific cell killing. Test different time frames.
• Check concentrations of stains and media additions. Titrate Cytotox Red and Caspase-3/7 Green reagents.
• Bacterial contamination. Check for a solution in problem 3.
• Conduct CS&T to establish if the detectors are functional. Contact the responsible technician if the cytometer is not performing accurately.

### Problem 7

No target cell killing or induction of apoptosis by suspected cytotoxic cells.

### Potential solution

• Too few effector cells were added to the well. Adjust the target-to-effector cell ratio, or carry out a cytotoxicity assay with various target-to-effector ratios. Ensure the accuracy of target and effector cell counts before the co-culture. See ‘Preparation of target cells’ and ‘Preparation of effector cells’.
• Effector cells are not being activated. Adjust the concentration of cytokines in the media that effector cells rely on. Ensure transgene expression on effectors and target antigen on targets.
• Certain target cells may be exceptionally resistant to killing by certain effector cells. Test with a range of target cells.
• It may be that effector cells are genuinely non-cytotoxic. Use a positive control (e.g., staurosporine).
• The time frame was too short. Test a range of incubation lengths.
• Check concentrations of stains and media additions. Increase Cytotox Red and Caspase-3/7 Green staining concentrations if they are too low.
• Dead cell or apoptotic cell stains are not functional. Attempt with fresh aliquots. Substitute Cytotox Red for propidium iodide to establish if this detects dead cells. Check a well by staining with trypan blue and counting dead cells on a hemacytometer. Visually inspect the wells. Compare untreated target-only controls with a well containing effector cells, which should certainly be positive. Dead and dying cells will be smaller with a less well-defined membrane.
• Voltages for the detection of stains that indicate cell death or apoptosis are set too low. Check the machine voltages as outlined in the solutions to ‘Problem 1’. See ‘Pilot assay and cytometer configuration’.
• Incorrect gate setting on the SSC FSC plot. Ensure to include the apparently healthy cells and the apparently dead cells in the SSC FSC gate. Do not include the debris. See Figure 4A.
• The incorrect detector was used. Analyze with all detectors enabled to determine which is best suited for that particular stain on that particular machine. See Figure S3.
• The cytometer cannot detect that particular stain. Test on another machine.
• Overcompensation. Try analyzing without compensation applied. Complete compensation again with new compensation controls if necessary. See ‘Prepare single stain controls for compensation’.
• Conduct CS&T to establish if the detectors are functional. Contact the responsible technician if the cytometer is not performing accurately.

### Problem 8

The calculated cell number is far higher or lower than expected.

### Potential solution

• Check calculations are accurate. See the ‘Target and effector cell preparation’ section for the calculation of cell numbers with beads.
• Check the concentration of count beads. Beads can be counted with a hemacytometer to confirm their concentration.
• The cell number was too high during setup. Ensure accuracy of counting when preparing cells for the cytotoxicity assay. Reduce cell seeding density. See the ‘Target and effector cell preparation’ section.
• Bacterial contamination. Check for the solution to problem 1.
• The calculated cell number is correct. Collect a 10 μL volume from the well after recording. Count using a hemacytometer and trypan blue to confirm.

### Problem 9

The recorded event number is too low.

### Potential solution

• Increase the number of cells in the wells during assay setup.
• Increase the volume available for acquisition. After the 1 h incubation of cells with counting beads, Caspase-3/7 Green, and Cytotox Red, add an additional volume of media. As the dead volume of media left in the well after acquisition remains constant regardless of the volume of media taken for analysis, a greater fraction of the cell suspension will be measured. This will substantially increase the number of recorded events without increasing the volume of counting beads, Caspase-3/7 Green, or Cytotox Red. This will, however, increase the time taken to record wells.

## Resource availability

### Lead contact

Further information and requests for resources and reagents should be directed to the lead contact, Prof. Gerald Wulf (gerald.wulf*@*med.uni-goettingen.de).

### Technical contact

Technical questions on executing this protocol should be directed to the technical contact, Kristian Thomson (kristian.thomson@med.uni-goettingen.de).

### Materials availability

This study generated no new unique reagents.

### Data and code availability

• Original data reported in this paper will be shared by the lead contact upon request.
• This paper does not report original code.
• Any additional information required to reanalyze the data reported in this paper is available from the lead contact upon request.

## Acknowledgments

The majority of this work was carried out in and supported by the University Medical Center Göttingen. The BD LSRFortessa X-20, used extensively in this protocol, was funded by the 10.13039/501100001659Deutsche Forschungsgemeinschaft (10.13039/501100001659DFG), project no. 442249343.

We would like to extend our utmost gratitude to the 10.13039/100019147UMG Flow Cytometry Core Facility for the opportunity to use its machines and for its outstanding expertise. We sincerely thank Miltenyi Biotec B.V. & Co. KG for supplying primary NK and CAR-NK cells and required reagents. We thank Dr. Denise Müller from the Department of Pathology and the Sartorius Corporate Research Department for their contribution to this work by supplying Cytotox viability stains. We would like to thank Dr. Julia Thomson for the critical evaluation of the manuscript before submission. We would like to thank Dr. Justin Hasenkamp for the initial suggestion to use flow cytometry for our cytotoxicity assays. Illustrations were created using https://BioRender.com.

## Author contributions

Conceptualization, K.T., G.W., and N.E.; experimental work, K.T., K.K., H.J., C.D., D.S., and M.Q.; data analysis, K.T., K.K., and H.J.; manuscript preparation (original draft), K.T.; manuscript revisions and editing, K.T., K.K., H.J., G.W., N.E., C.D., D.S., and M.Q.; supervision, G.W., J.W., and R.K.; resources, G.W., J.W., C.D., D.S., and M.Q.; funding acquisition, G.W.

## Declaration of interests

C.D., D.S., and M.Q. are employees of Miltenyi Biotec B.V. & Co. KG. Miltenyi Biotec B.V. & Co. KG supplied WT NK and CAR-NK cells and NK cell culture reagents as part of an ongoing collaboration. The Sartorius Corporate Research Department supplied samples of Cytotox Red and Cytotox NIR as part of an ongoing collaboration.

## Declaration of generative AI and AI-assisted technologies in the writing process

During the preparation of this work, the authors used ChatGPT (OpenAI) in order to generate code for use in the statistical software environment “R”. Spelling and grammar were checked using Grammarly. After using these tools/services, the authors reviewed and edited the content as needed and take full responsibility for the content of the published article.
