# Supplementary material for: Simultaneous measurement of target and effector cell death, apoptosis, and proliferation in cell therapy development using flow cytometry
Source: STAR Protoc. 2026 Jul 23;7(3):104703. doi: 10.1016/j.xpro.2026.104703 (PMC13427446; doi:10.1016/j.xpro.2026.104703)
Supplement: Document S1. Figures S1–S19 and Tables S1–S8 [file mmc1.pdf]

Supplementary

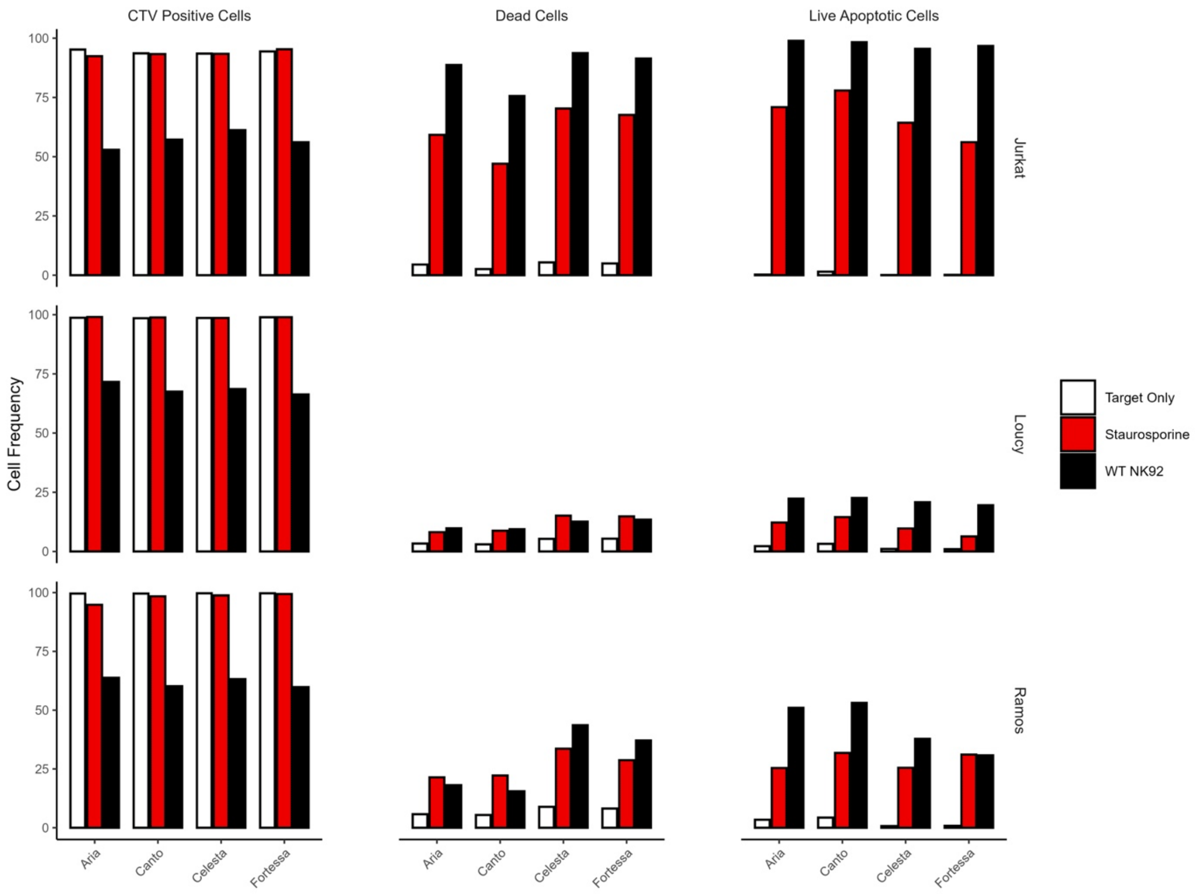

Figure S1. Results generated using the staining panel are comparable between various flow cytometers, related to step 4 of "Pilot assay and cytometer configuration". Jurkat, Loucy, and Ramos cells were stained with 100 µL DPBS containing 10 µM CTV for 20 min at 37°C. Cells were washed appropriately and cultured for 24 h at 37°C in the presence of either 0.1 µM staurosporine or a 1:1 target to effector ratio of WT NK92 cells or left untreated. Cells were analyzed on four different flow cytometers with distinct laser and detector configurations. Dead cells were detected using Cytotox Red, and apoptotic cells were detected using Caspase-3/7 Green. Fluorescent beads, cellular debris, and doublets were excluded using the gating strategy depicted in Figure 4. Data are representative of a single biological replicate.

Supplementary

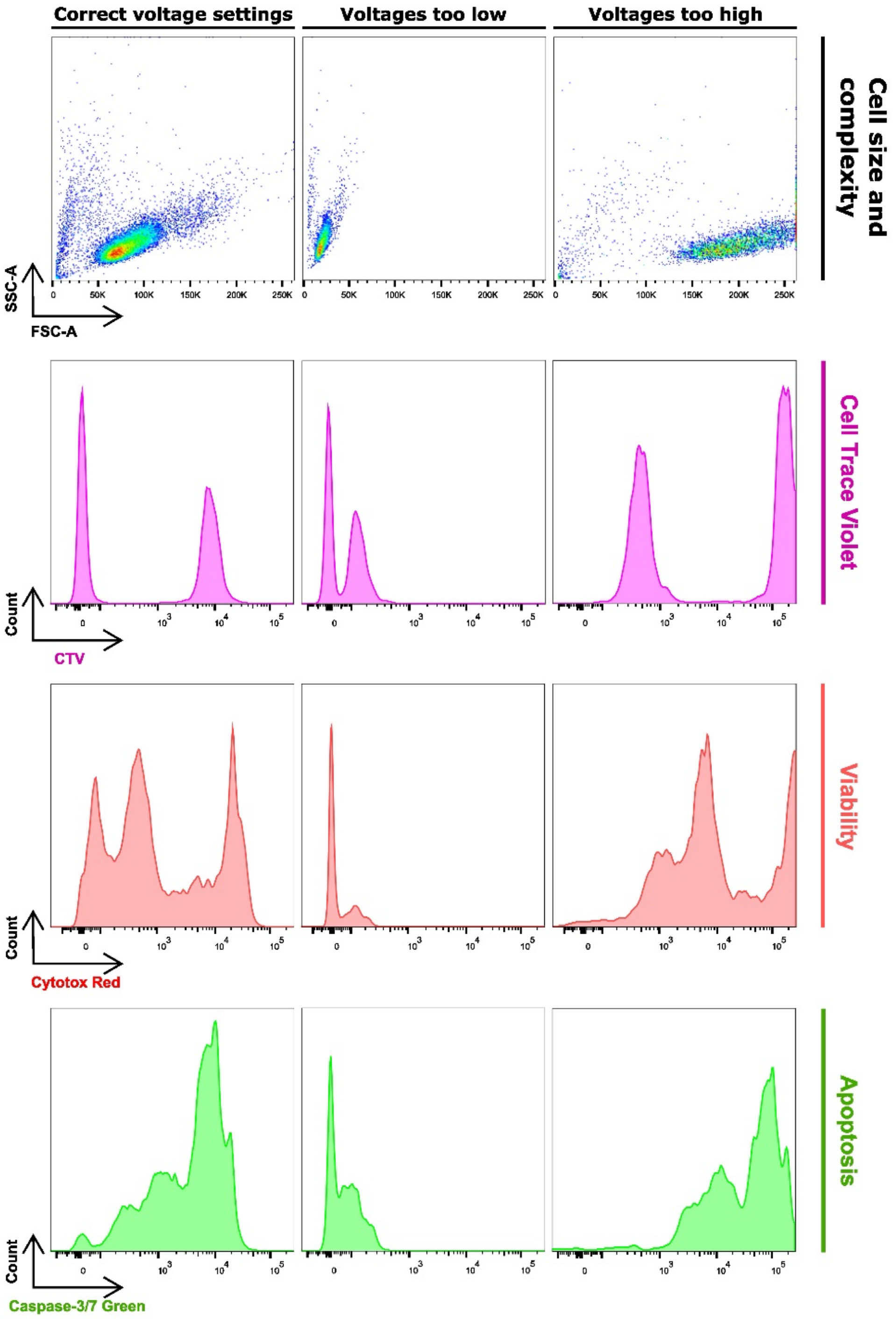

Figure S2. Setting detector voltages, related to Step 4 of "Pilot assay and cytometer configuration". For the CTV control, Jurkat cells were stained with 100 µL PBS containing 10 µM CTV for 20 min at 37°C with agitation at 10 min. After washing, CTV-positive cells were incubated for 24 h at 37°C. Unstained Jurkat cells were added, and cells were analyzed. For the Cytotox Red and Caspase-3/7 Green single stain controls, unstained Jurkat cells were treated with 0.1 µM staurosporine. After 24 h, a small volume of untreated cells was added. Separately, cells were stained with either a 1:7,000 dilution of Cytotox Red or a 1:1,000 dilution of Caspase-3/7 Green for 1 h at 37°C in the dark. Cells were then analyzed. Ungated cells are depicted.

Supplementary

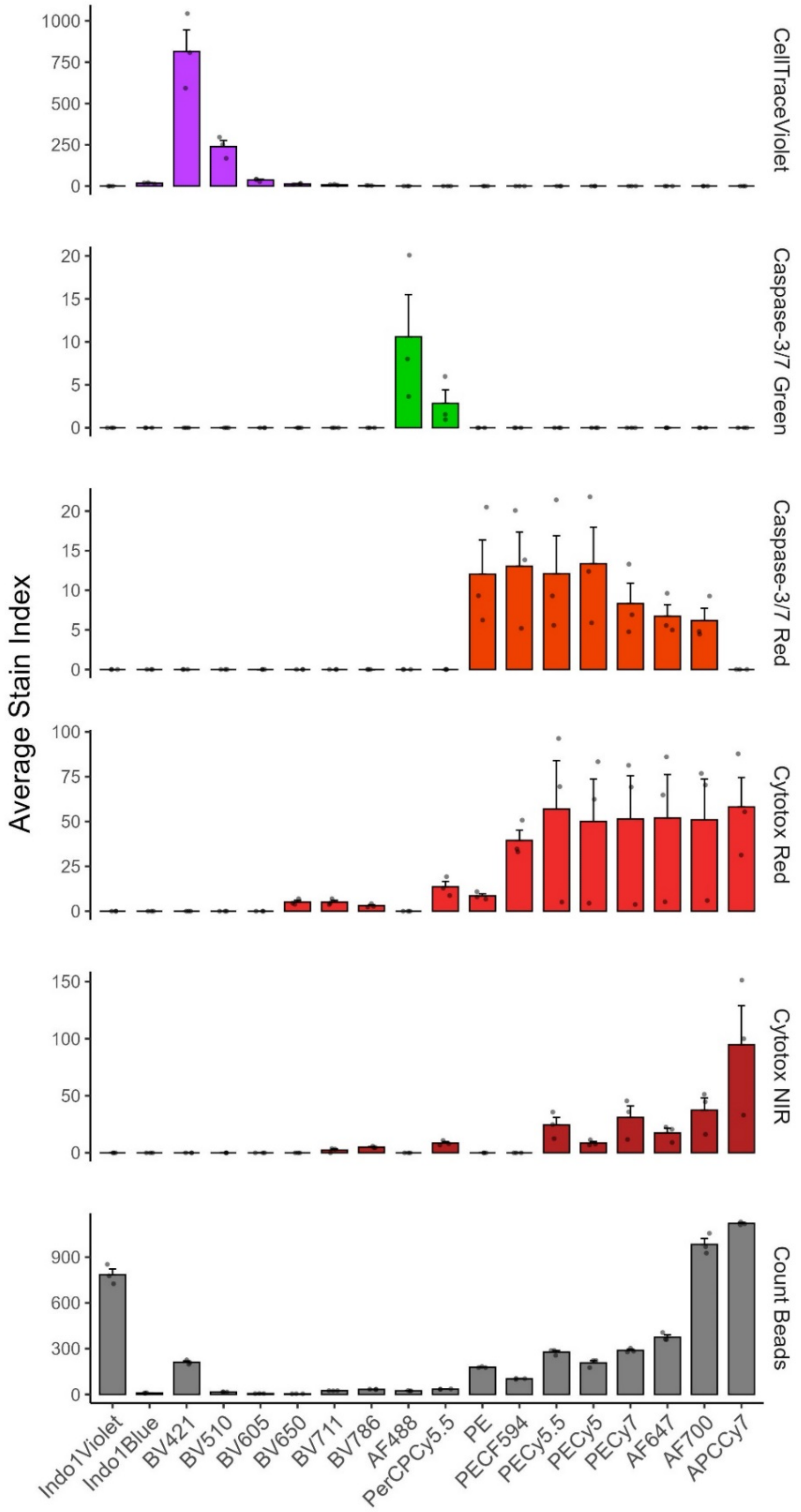

Figure S3. Stain indexes of CellTrace Violet, Caspase-3/7 Green and Red, Cytotox Red and NIR, and fluorescent counting beads on an LSRFortessa X-20, related to Step 5 of "Pilot assay and cytometer configuration". For the counting beads, unstained cells were used as the negative populations. Values from detectors that resulted in no clearly distinguishable positive populations were normalized to zero. Data points represent the mean calculated stain index of three different cell lines: Jurkat, Loucy, and Ramos. Error bars represent SEM. For the detection of Caspase-3/7 and viability, cells were incubated for 24 h at 37°C with 0.1 µM staurosporine.

Supplementary

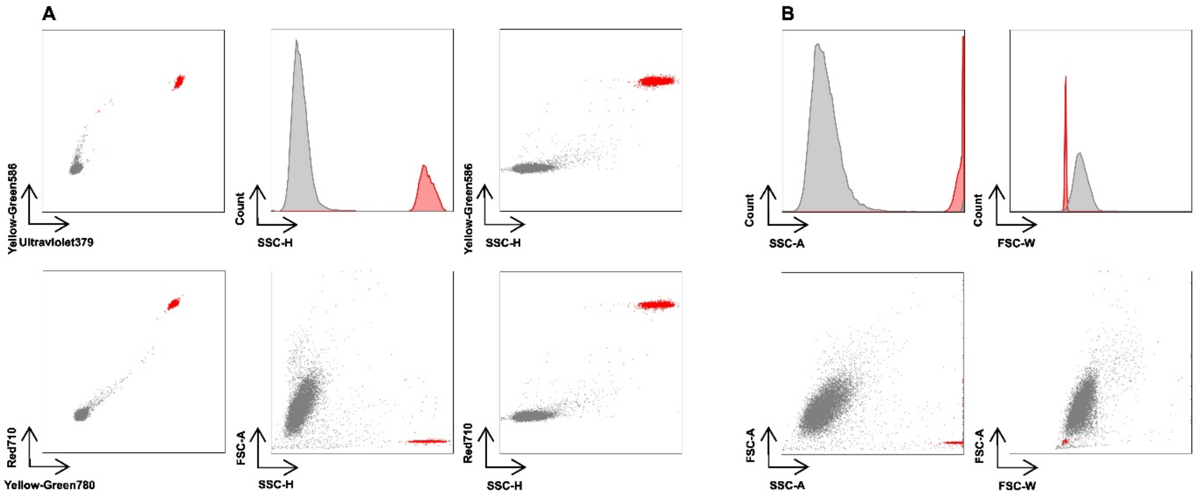

Figure S4. Fluorescent counting bead detection, related to Step 5 of "Pilot assay and cytometer configuration". A) Examples of strategies that can be employed to identify bead populations. B) Examples of strategies that should not be used to identify bead populations. Unlabeled Jurkat cells mixed with fluorescent count beads and analyzed on a BD LSRFortessa X-20. Grey indicates Jurkat cells. Red indicates the fluorescent bead population.

Supplementary

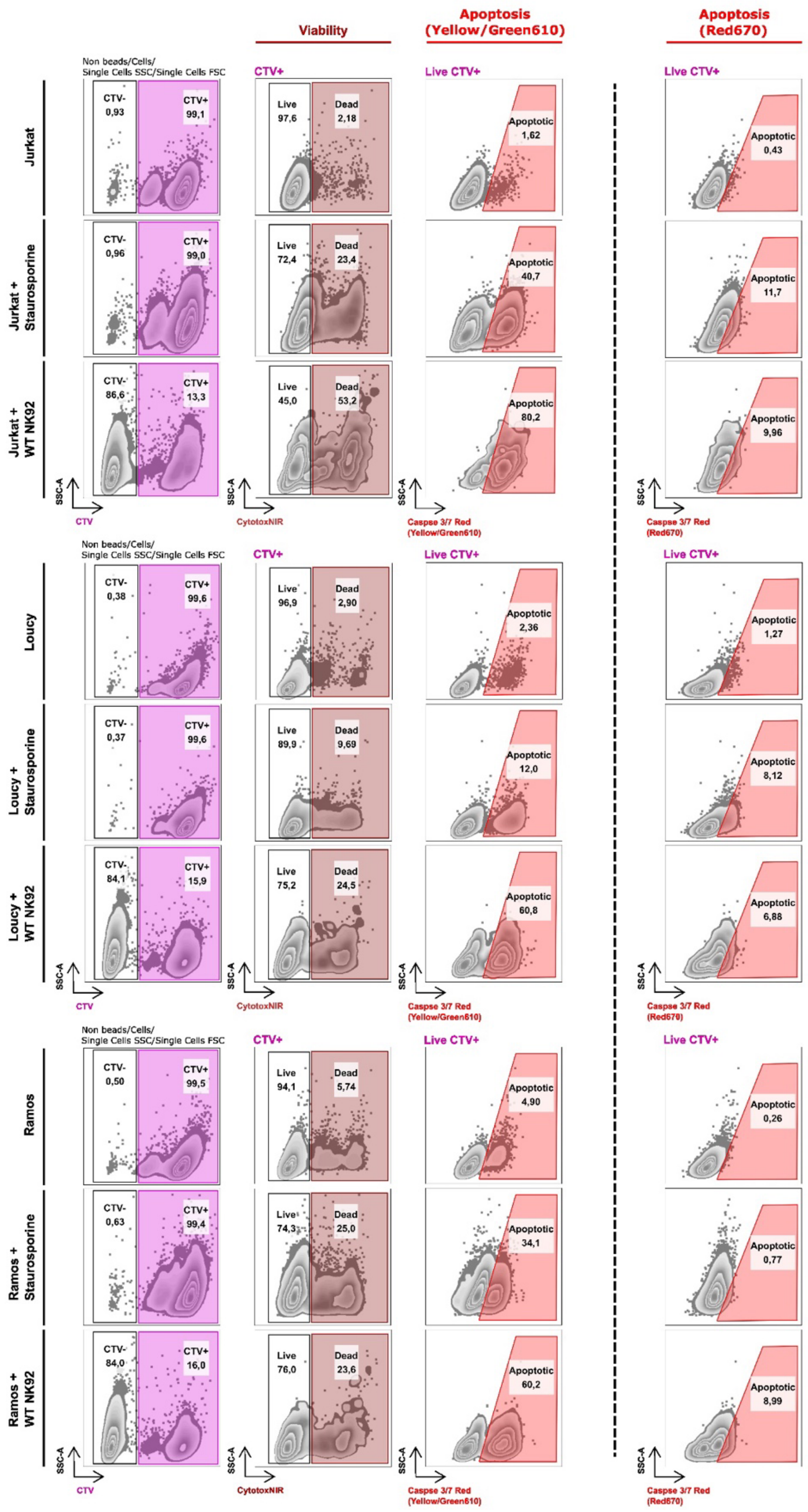

Figure S5. CTV, Cytotox NIR, and Caspase-3/7 Red fluorescent signals can be detected in GFP-positive CTV-stained Jurkat, Loucy, and Ramos cells, and used to identify target, dead, and apoptotic cells, related to Step 5 of "Pilot assay and cytometer configuration". GFP-expressing CTV-stained Jurkat, Loucy, and Ramos cells were cultured for 24 h at 37°C with or without 0.1 µM staurosporine, or WT NK92 cells (target to effector ratio of 1:1). Dead cells were detected using Cytotox NIR, and apoptotic cells were detected using Caspase-3/7 Red with a yellow-green laser and a 610/20 detector or a red laser and a 670/30 detector.

Supplementary

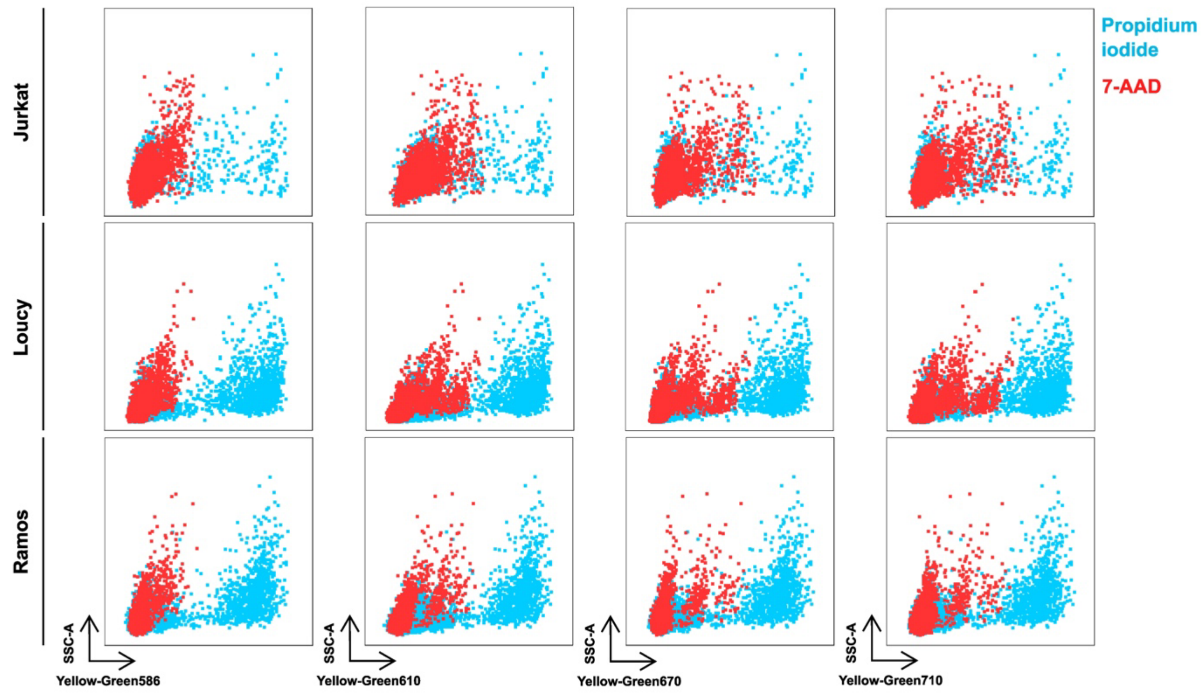

Figure S6. Propidium iodide provides a bright signal useful for live/dead cell discrimination, related to Step 5 of "Pilot assay and cytometer configuration". Jurkat, Loucy, and Ramos cells were stained with 0.5 µg/mL propidium iodide or 0.525 µg/mL 7-AAD and analyzed using a yellow-green laser with 586/15, 610/20, 670/30, and 710/50 detectors. Cellular debris and doublets were excluded prior to analysis.

Supplementary

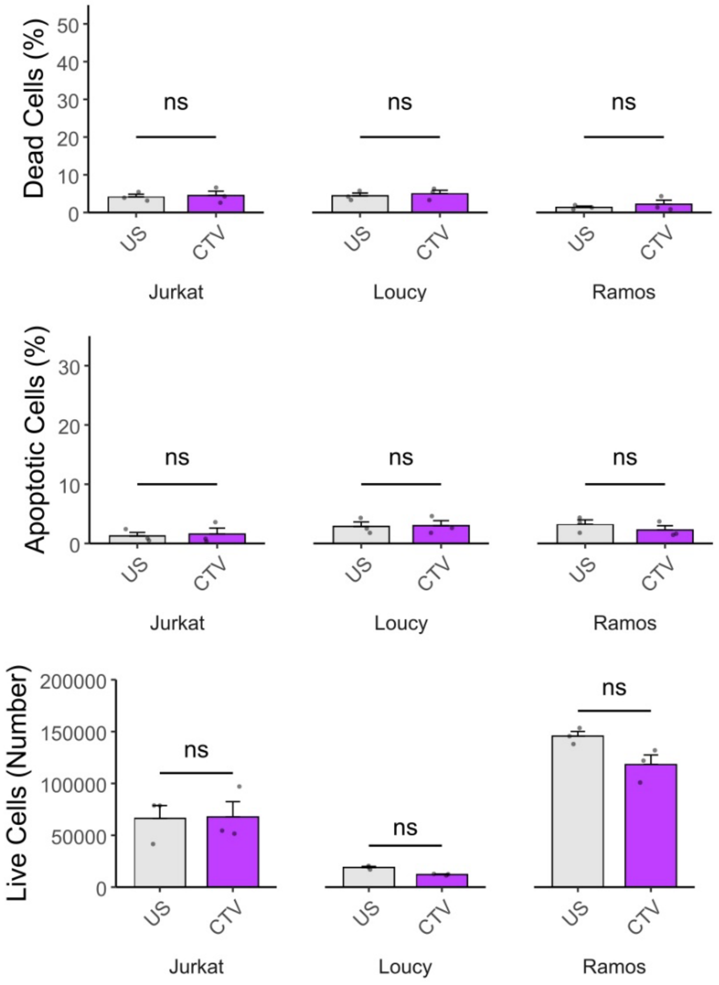

Figure S7. CTV-labeled Jurkat, Loucy, and Ramos cell death, apoptosis, and number are similar to non-stained controls after culture for 72 h, related to Step 1 of "Target and effector cell preparation". 1 × 10^6 Jurkat, Loucy, and Ramos cells were stained with 100 μL DPBS containing 10 μM CTV for 20 min at 37°C. Cells were washed and cultured for 72 h at 37°C. Dead cells were detected with Cytotox Red, and apoptotic cells were detected with Caspase-3/7 Green. Fluorescent count beads were used to determine cell numbers. Cellular debris and doublets were excluded before analysis. Two-sided Wilcoxon test (ns = not significant). n = 3. Data are represented as mean + SEM.

Supplementary

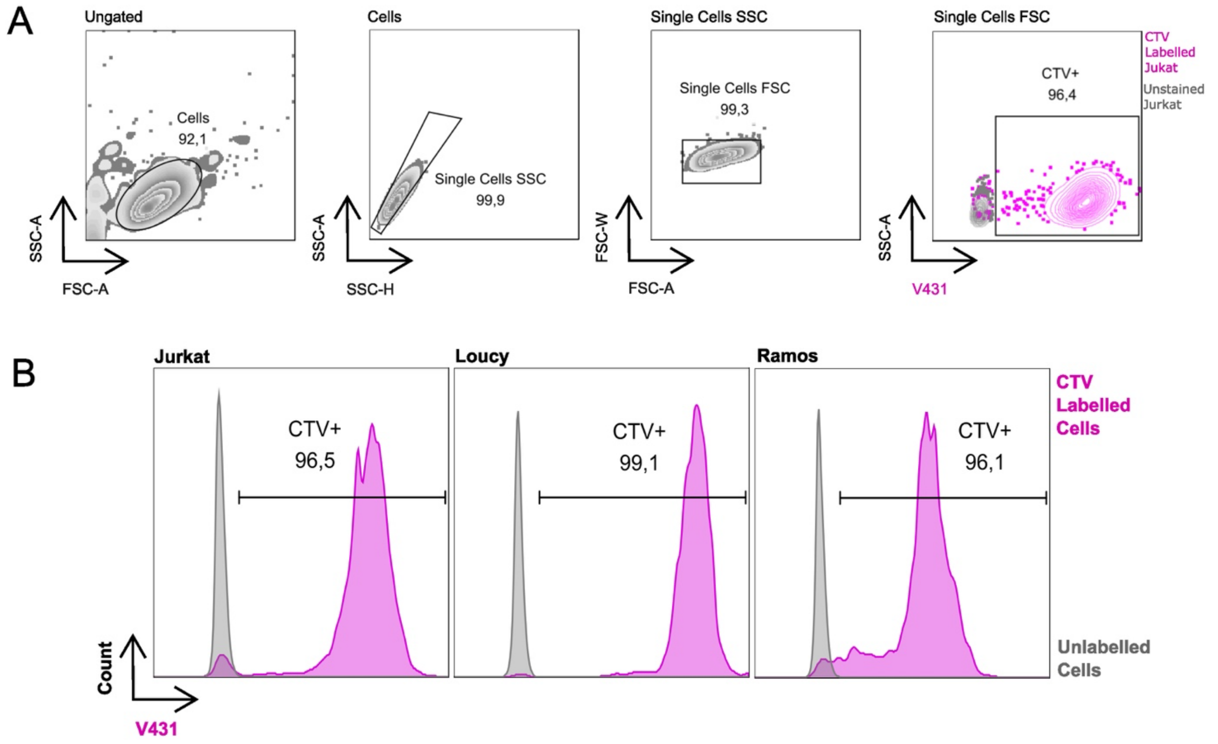

Figure S8. CellTrace Violet signal can be detected in Jurkat, Loucy, and Ramos cells 72 h after staining, related to Step 1 of "Target and effector cell preparation". 1 × 10^6 Jurkat, Loucy, and Ramos cells were stained with 100 µL PBS containing 10 µM CTV for 20 min at 37°C with agitation at 10 min, or left unstained. Cells were washed appropriately and cultured for 72 h at 37°C. A) Gating strategy used to identify CTV-negative and CTV-positive cells. CTV-positive or -negative Jurkat cells are depicted here as an example. B) Purple histograms indicate CTV-stained cells. Grey histograms depict unstained cells. CTV-positive cell frequency of single-cell events is depicted. Cellular debris and doublets were excluded using the gating strategy. Even after 72 h, more than 95% of target cells remain CTV-positive.

Supplementary

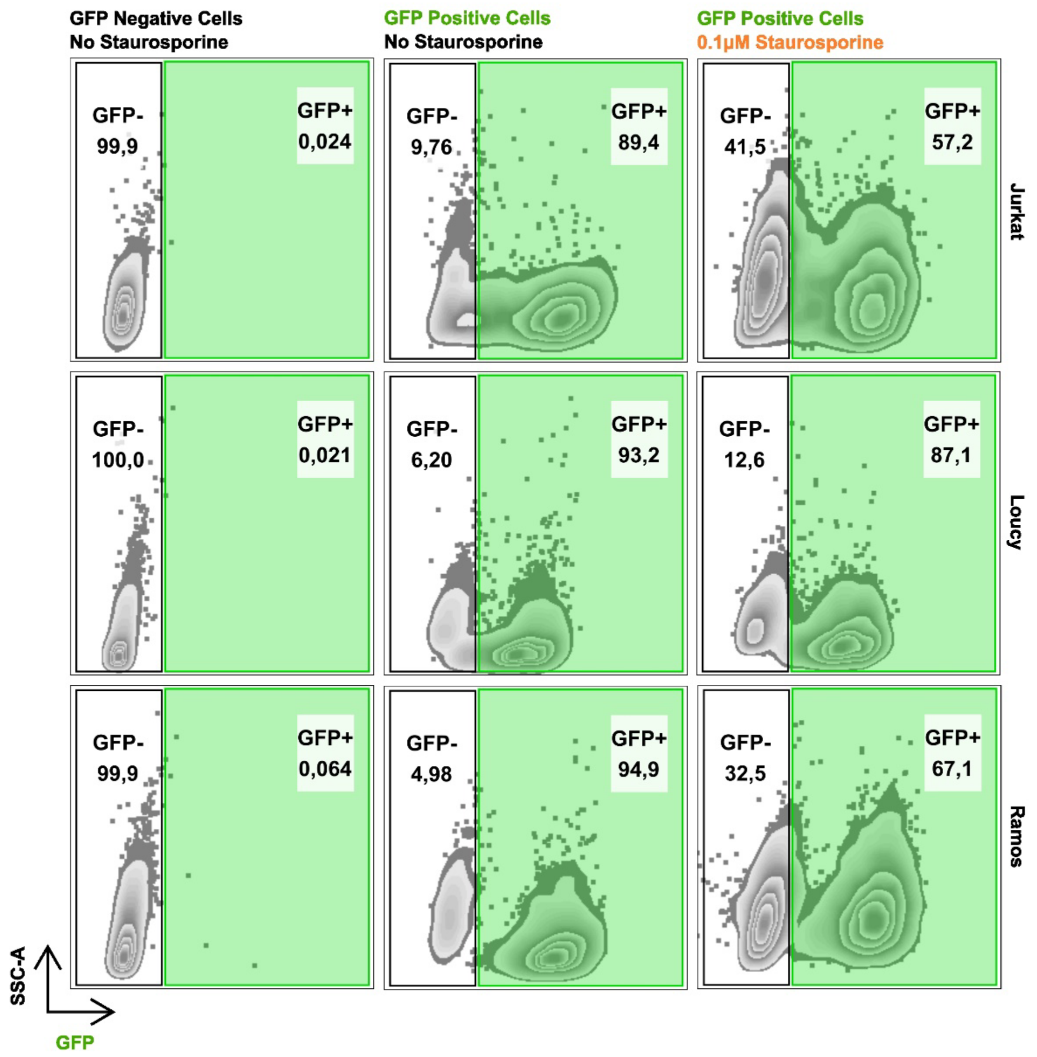

Figure S9. GFP signal is reduced in Jurkat, Loucy, and Ramos cells after exposure to staurosporine, related to Step 1 of "Target and effector cell preparation". WT or GFP expressing Jurkat, Loucy, and Ramos cells were cultured for 24 h at 37°C with or without 0.1 μM staurosporine. Cellular debris and doublets were excluded during analysis.

Supplementary

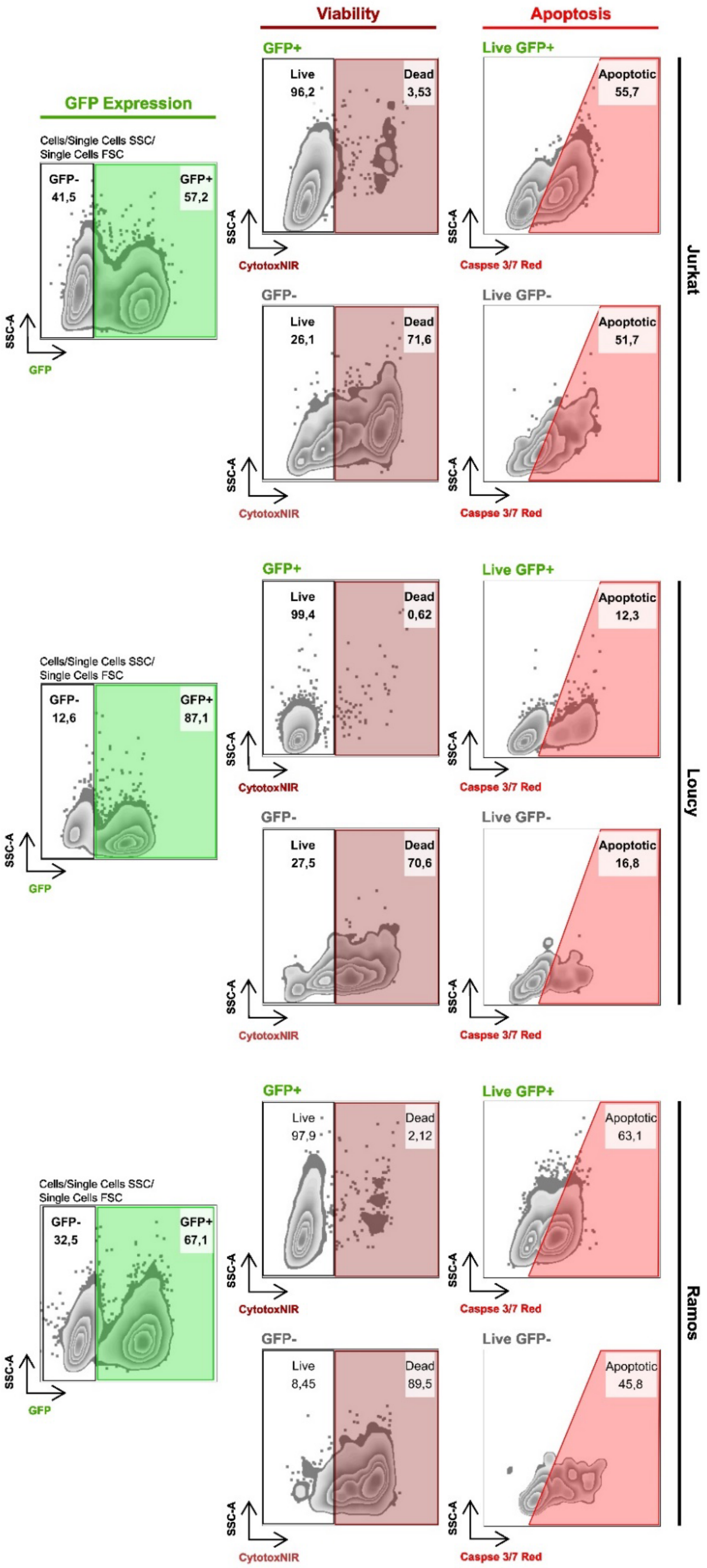

Figure S10. GFP signal, dead cell, and apoptotic cell detection in GFP-expressing Jurkat, Loucy, and Ramos cells after exposure to staurosporine, related to Step 1 of "Target and effector cell preparation". GFP-expressing Jurkat, Loucy, and Ramos cells were cultured for 24 h at 37°C with or without 0.1 μM staurosporine. Dead cells were detected using Cytotox NIR, and apoptotic cells were detected using Caspase-3/7 Red. Cellular debris and doublets were excluded using the gating strategy.

Supplementary

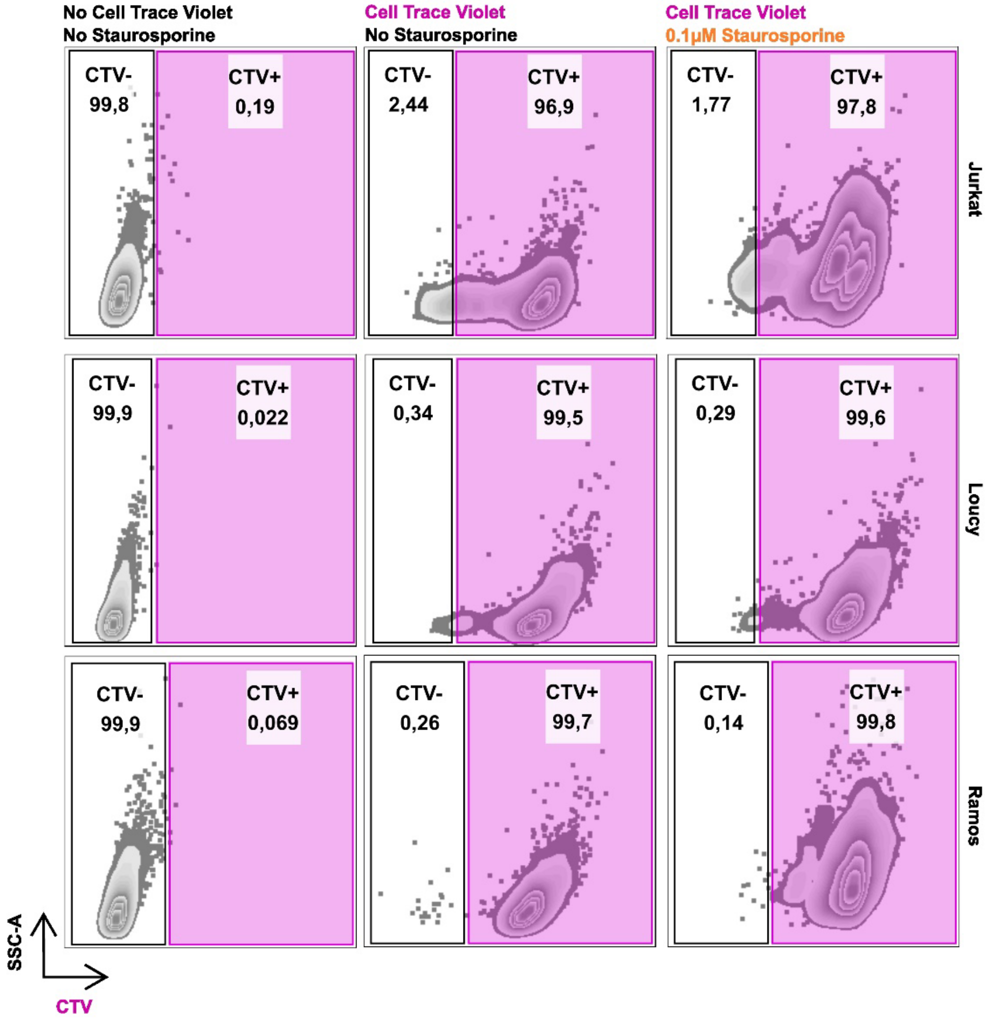

Figure S11. CTV signal is not reduced in Jurkat, Loucy, and Ramos cells after exposure to staurosporine, related to Step 1 of "Target and effector cell preparation". Jurkat, Loucy, and Ramos cells were stained with 100 μL DPBS containing 10 μM CTV for 20 min at 37°C or left unlabeled. Cells were washed appropriately and cultured for 24 h at 37°C with or without the addition of 0.1 μM staurosporine. Fluorescent beads, cellular debris, and doublets were excluded during analysis.

Supplementary

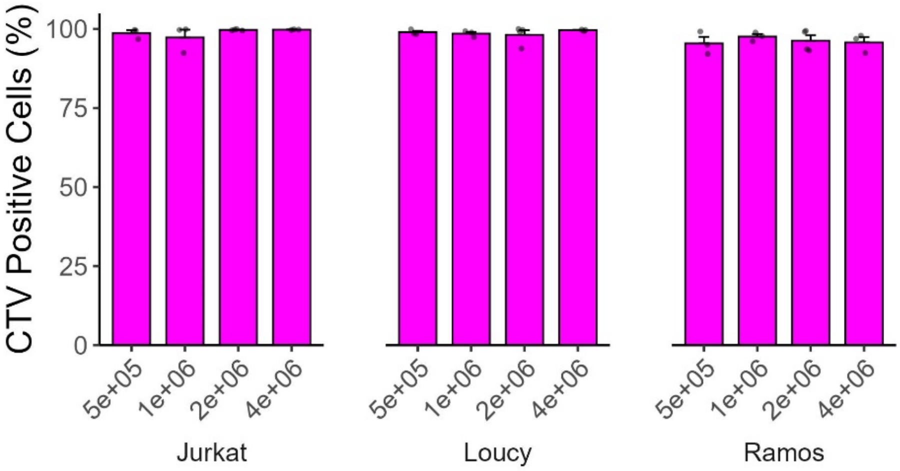

Figure S12. CellTrace Violet staining efficiency, related to Step 1 of "Target and effector cell preparation". Between 5 × 10^5 and 4 × 10^6 Jurkat, Loucy, and Ramos cells were stained with 100µL PBS containing 10 µM CTV for 20 min at 37°C with agitation at 10 min. CTV-positive cell frequency of single-cell events is shown. Cellular debris and doublets were excluded using the gating strategy. Data are represented as mean + SEM.

Supplementary

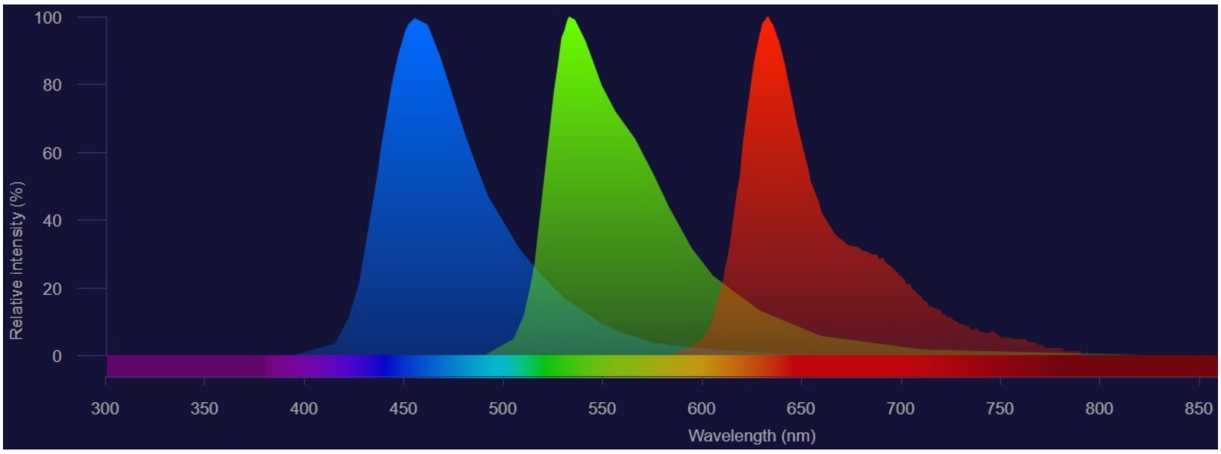

Figure S13. Spectral emission curves for CellTrace Violet (blue), Caspase-3/7 Green (green), and YOYO-3 (red histogram), related to Step 2 of "Target and effector cell preparation". YOYO-3, a fluorochrome with similar excitation and emission properties to those of Cytotox Red, is used here in place of Cytotox Red. An interactive Cytotox Red emission histogram is not available online. Histograms were created using the fluorescence SpectraViewer from ThermoFisher.

Supplementary

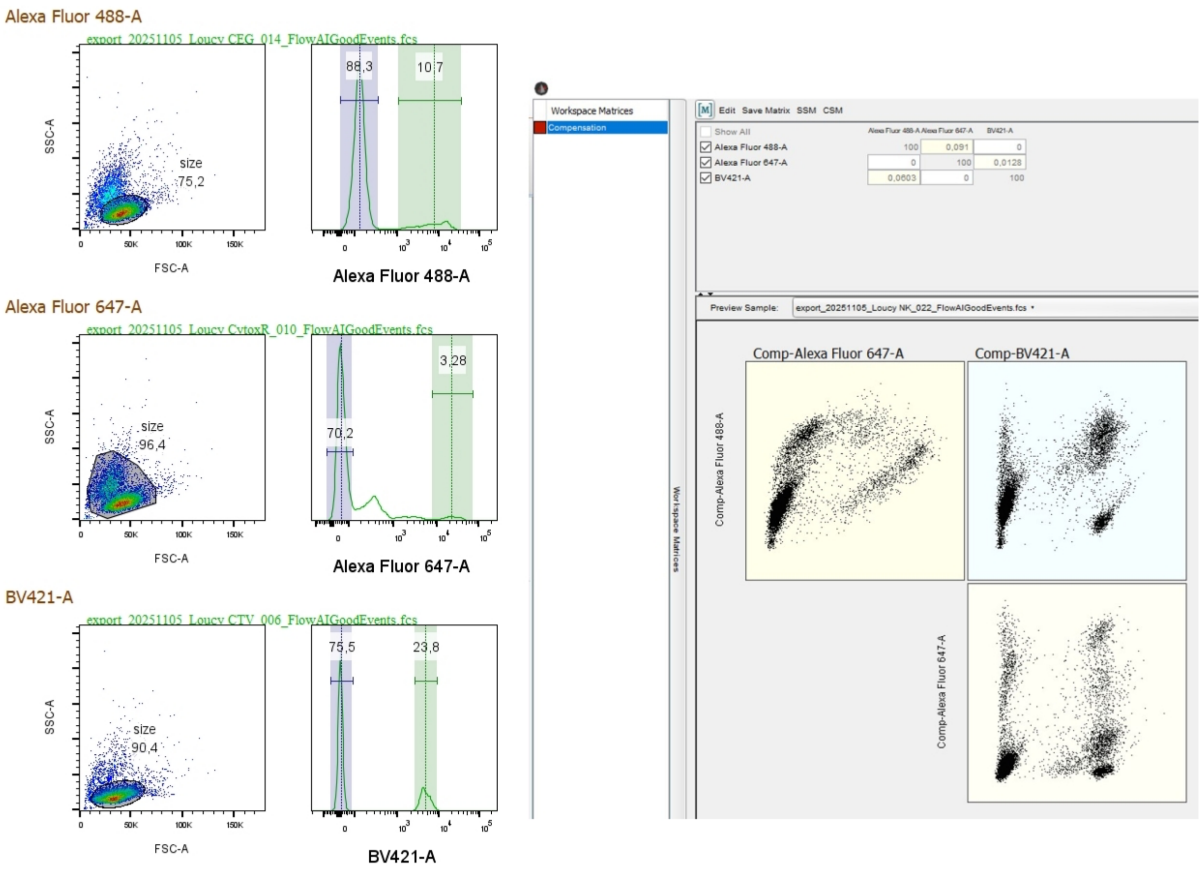

Figure S14. Compensation controls and compensation matrix example, related to Step 2 of "Target and effector cell preparation". For the CTV single-stain control, Loucy cells were stained with 100 µL PBS containing 10 µM CTV for 20 min at 37°C, with agitation every 10 min. After washing, CTV-positive cells were incubated for 24 h at 37°C. Unstained Loucy cells were added, and cells were analyzed. For the Cytotox Red and Caspase-3/7 Green single stain controls, unstained Loucy cells were treated with 0.1 µM staurosporine. After 24 h, a small volume of untreated cells was added. Separately, cells were stained with either a 1:7,000 dilution of Cytotox Red or a 1:1,000 dilution of Caspase-3/7 Green for 1 h at 37°C in the dark.

Supplementary

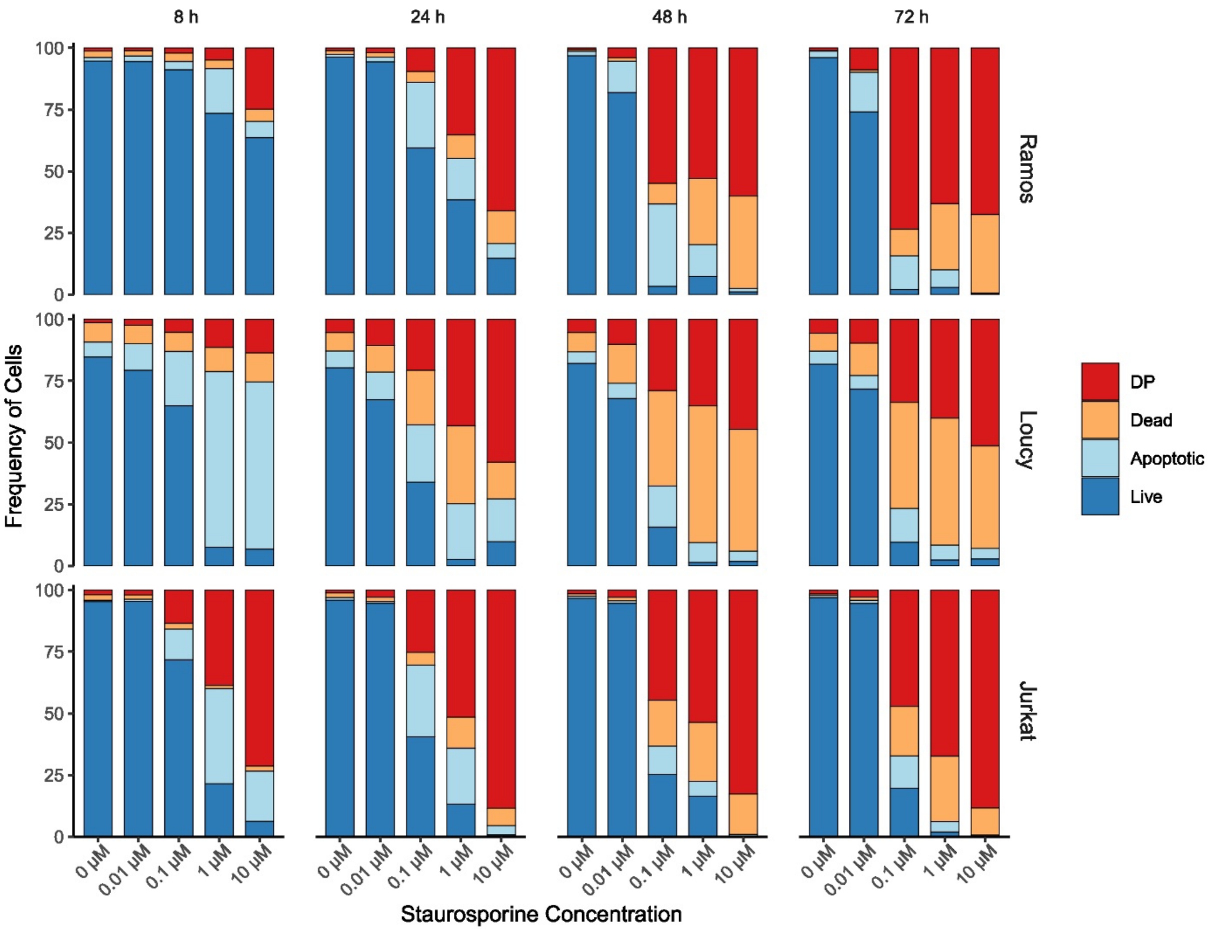

Figure S15. Staurosporine time course and dose response, related to Step 2 of "Target and effector cell preparation". Jurkat, Loucy, and Ramos cells were treated with various concentrations of staurosporine for 8, 24, 48, and 72 h. Dead cells and apoptotic cells were detected using Cytotox Red and Caspase-3/7 Green, respectively. Mean cell frequencies of three independent experiments are shown. DP (double-positive) represents cells that are positive for both Cytotox Red and Caspase-3/7 Green.

Supplementary

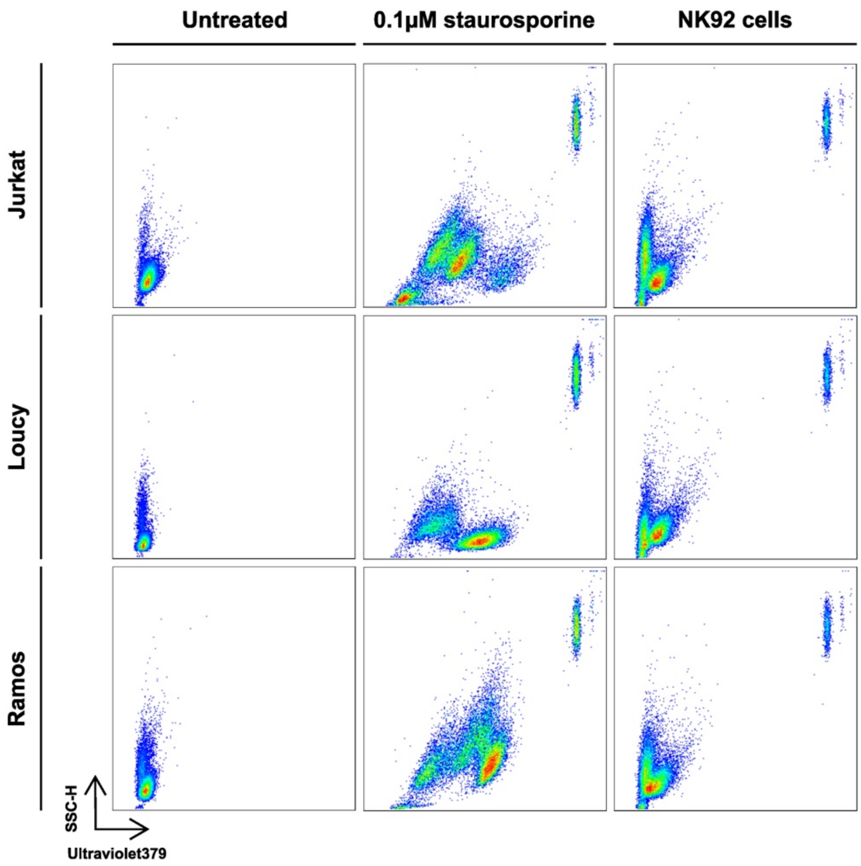

Figure S16. Autofluorescence in the ultraviolet spectrum with staurosporine treatment, related to Step 2 of "Target and effector cell preparation". Jurkat, Loucy, and Ramos cells were stained with 100 μL PBS containing 10 μM CTV for 20 min at 37°C with agitation at 10 min. After washing, CTV-positive cells were incubated for 24 h at 37°C in the presence of 0.1 μM staurosporine, a 1:1 ratio of WT NK92 cells, or left untreated. Fluorescent counting beads were added, and samples were then analyzed for their fluorescence. Ungated cells are depicted.

Supplementary

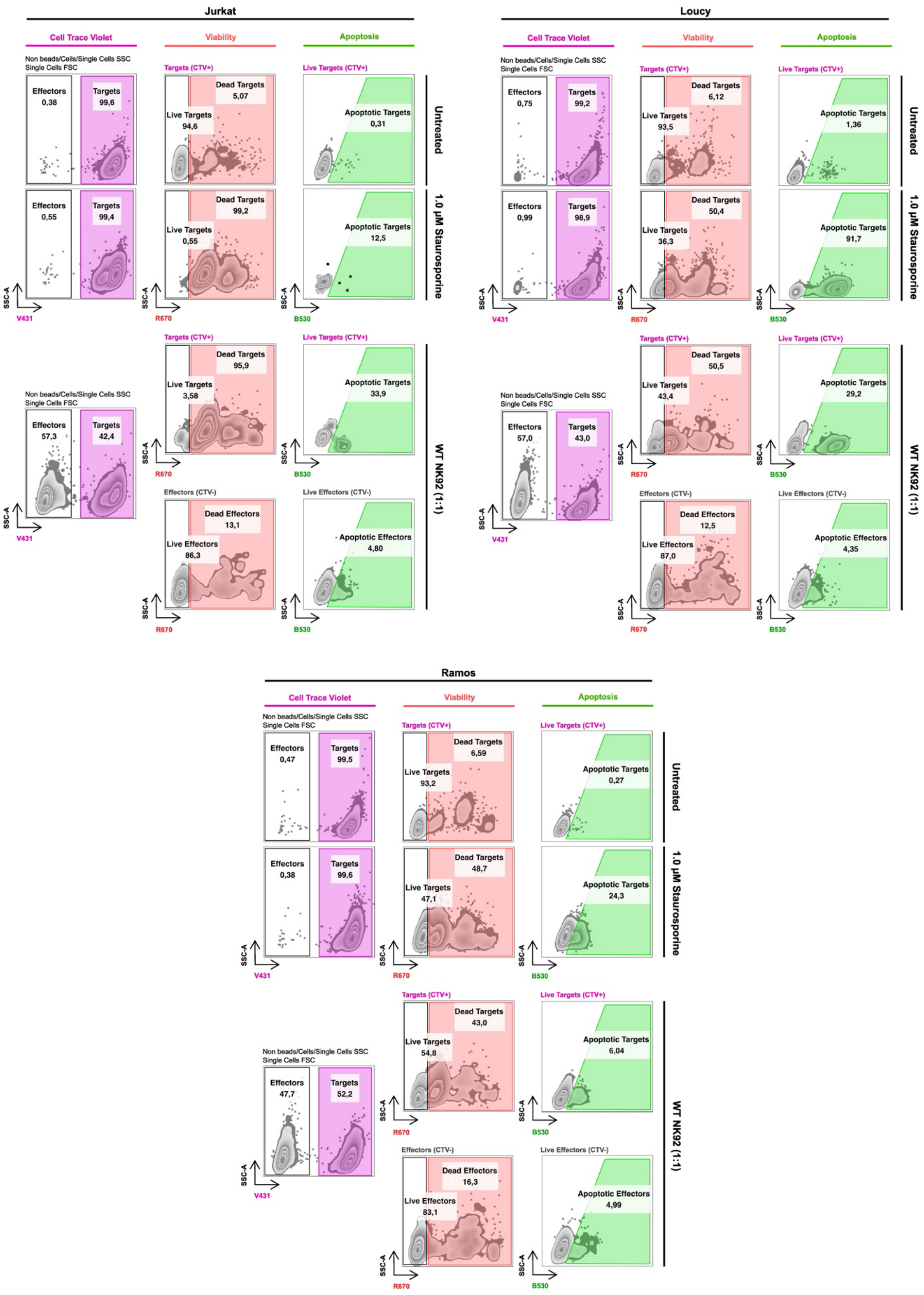

Figure S17. Same-day detection of cytotoxicity, related to Step 3 of "Target and effector cell preparation". Jurkat, Loucy, and Ramos cells were stained with 100 µL DPBS containing 10 µM CTV for 20 min at 37°C. After washing, target cells, staurosporine (end concentration of 1 µM), or WT NK92 cells (target to effector ratio of 1:1) were added to the appropriate wells of a 96-well round-bottom plate. The plate was centrifuged at 300 × g at RT for 3 min, and the cells were then cultured for 8 h at 37°C. Dead cells were detected using Cytotox Red, and apoptotic cells were detected using Caspase-3/7 Green.

Supplementary

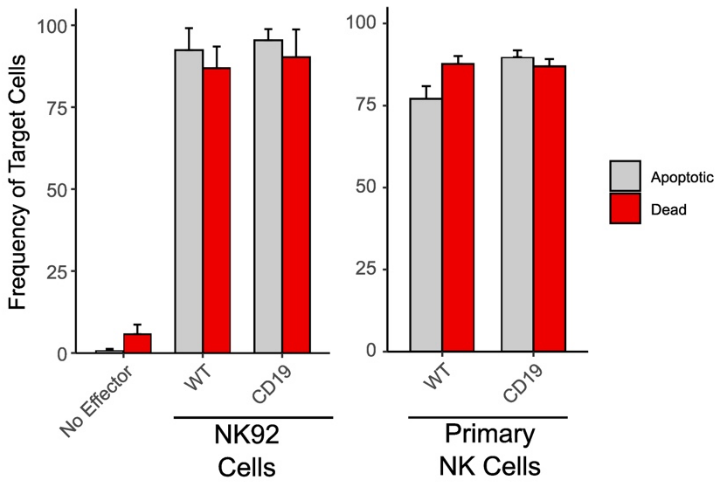

Figure S18. Jurkat cells are sensitive to innate killing by WT NK92 and primary WT NK cells, related to Step 3 of "Target and effector cell preparation". Jurkat cells were stained with 100 µL DPBS containing 10 µM CTV for 20 min at 37°C. After washing, target and effector cells at a ratio of 1:1 were added to the appropriate wells of a 96-well round-bottom plate. Cells were incubated for 24 h at 37°C. Dead cells were detected using Cytotox Red, and apoptotic cells were detected using Caspase-3/7 Green. n = 2-4. Data are represented as mean + SEM.

Supplementary

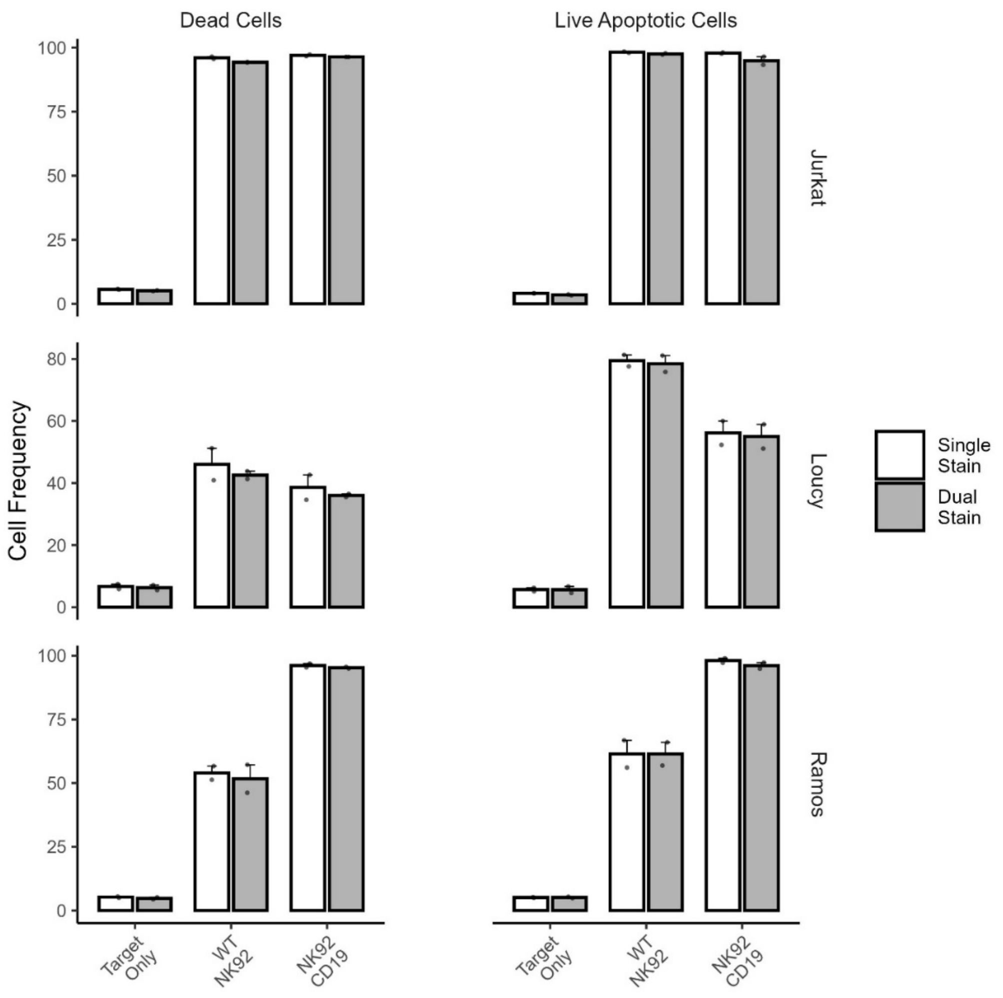

Figure S19. The frequency of dead and apoptotic cells is comparable between single-stained and dual-stained CTV-positive Jurkat, Loucy, and Ramos cells after incubation with effectors, related to Step 4 of "Analysis of cytotoxicity assay". Target cells were stained with 100 µL PBS containing 10 µM CTV for 20 min at 37°C with agitation at 10 min. Cells were washed appropriately and cultured for 48 h at 37°C with or without a 1:1 target-to-effector ratio of WT NK92 cells or anti-CD19 CAR-expressing NK92 cells. Dead cells were detected using Cytotox Red, and apoptotic cells were detected using Caspase-3/7 Green, either alone or in combination. Cellular debris and doublets were excluded prior to analysis. Frequency Cytotox Red or Caspase-3/7 Green positive cells of CTV positive events are shown. n = 2. Data are represented as mean + SEM.

Supplementary

Table S1. BD LSRFortessa™ X-20 configuration used to establish the current protocol, related to Step 1 of "Pilot assay and cytometer configuration".

LASER	FILTER	REAGENTS USED IN THIS PROTOCOL	EXAMPLE FLUOROPHORES
355 nm	379/28	Fluorescent counting beads	BUV395, Indo-1 (Violet)
	515/30		BUV496, Indo-1 (Blue)
405 nm	431/28	CTV	BV421, Pacific Blue, VioBlue
	525/50		BV510, VioGreen
	610/20		BV605, Vio Bright V600
	670/30		BV650, Vio Bright V646
	710/50		BV711
	780/60		BV786
488 nm	488/10 (SSC)		N/A
	530/30	Caspase-3/7 Green, GFP	FITC, AF488
	710/50		PerCP-Cy5.5, BB700
561 nm	586/15	Fluorescent counting beads	PE
	610/20	Caspase-3/7 Red	PE-CF594, PE-Texas Red
	670/30	Propidium iodide, 7-AAD	PE-Cy5
	710/50		PE-Cy5.5
	780/60		PE-Cy7
637 nm	670/30	Cytotox Red	APC, AF647
	710/50		AF700
	780/60	Cytotox NIR	APC-Cy7

Table S2. BD FACSCelesta™, related to Step 1 of "Pilot assay and cytometer configuration".

LASER	FILTER	EXAMPLE FLUOROPHORES
405nm	450/40	BV421, Pacific Blue, VioBlue, **CellTrace Violet**[a]
	525/50	BV510, VioGreen
	610/20	BV605, Vio Bright V600
	670/30	BV650, Vio Bright V646
	780/60	BV786
488nm	488/10 (SSC)	Side scatter
	530/30	FITC, AF488, GFP[a], **CellEvent Caspase-3/7 Green**[a]
	575/25	PE
	610/20	PE-CF594, PE-Texas Red, propidium iodide[a]
	695/40	PE-Cy5, 7-AAD[a]
640nm	670/30	APC, AF647, **Cytotox Red**
	730/45	AF700
	780/60	APC-Cy7, Cytotox NIR[a]

[a]Used in this protocol and should be excited by the indicated laser and detected with the indicated filter.

Supplementary

Table S3. BD FACSAria™ II, related to Step 1 of "Pilot assay and cytometer configuration".

LASER	FILTER	EXAMPLE FLUOROPHORES
375nm	450/40	BV421, Pacific Blue, VioBlue, **CellTrace Violet**[a]
	670LP	BV650, Vio Bright V646, BV711, BV786
488nm	488/10 (SSC)	Side scatter
	530/30	FITC, AF488, GFP[a], **CellEvent Caspase-3/7 Green**[a]
	585/42	PE
	616/23	PE-CF594, PE-Texas Red, propidium iodide[a]
	695/40	PE-Cy5, 7-AAD[a]
	780/60	PE-Cy7
633nm	660/20	APC, AF647, **Cytotox Red**
	780/60	APC-Cy7, Cytotox NIR[a]

[a]Used in this protocol and should be excited by the indicated laser and detected with the indicated filter.

Table S4. BD FACSCanto™ II, related to Step 1 of "Pilot assay and cytometer configuration".

LASER	FILTER	EXAMPLE FLUOROPHORES
405nm	450/40	BV421, Pacific Blue, VioBlue, **CellTrace Violet**[a]
	585/29	BV570, VioGreen
488nm	488/10 (SSC)	Side scatter
	530/30	FITC, AF488, GFP[a], **CellEvent Caspase-3/7 Green**[a]
	585/42	PE
	670LP	PE-Cy5, PerCP, propidium iodide[a], 7-AAD[a]
	780/60	PE-Cy7
633nm	660/20	APC, AF647**, Cytotox Red**
	780/60	APC-Cy7, Cytotox NIR[a]

[a]Used in this protocol and should be excited by the indicated laser and detected with the indicated filter.

Table S5.CTV panel, related to Step 5 of "Pilot assay and cytometer configuration".

FUNCTION:	TARGET CELL IDENTIFICATION	VIABILITY	APOPTOSIS
Stain:	CellTrace Violet	Cytotox Red[a]	CellEvent Caspase-3/7 Green
Laser (filter):	405 nm (431/28)	637 nm (670/30)	488 nm (530/30)
Example stains:	Pacific Blue, BV421	APC, AF647	FITC, AF488

[a]Can also be detected adequately in PE-CF594, PE-Cy5.5, PE-Cy7, AF700, and APC-Cy7 channels.

Supplementary

Table S6. GFP-CTV panel, related to Step 5 of "Pilot assay and cytometer configuration".

FUNCTION:	TARGET CELL IDENTIFICATION	VIABILITY	APOPTOSIS
Stain:	CellTrace Violet	Cytotox NIR	CellEvent Caspase-3/7 Red[a,b]
Laser (filter):	405 nm (431/28)	637 nm (780/60)	561 nm (610/20)
Example stains:	Pacific Blue, BV421	APC-Cy7, APC-eFluor 780	PE-CF594, PE Texas red

[a]Can also be detected in PE, PE-Cy5, and PE-Cy5.5 channels.

[b]Must be excited by a yellow green laser. Signal cannot be distinguished with a red laser when in combination with Cytotox NIR.

Table S7. Comparison of hemacytometer vs bead-based counting method, related to Step 1 of "Target and effector cell preparation".

TECHNIQUE:	HAEMOCYTOMETER	BEAD BASED
Category:	Gold standard	Modern technique
Relative ease:	Easy	Moderate
Throughput – low sample numbers	Very fast	Very slow
Throughput – high sample numbers	Very slow	Fast
Cells counted:	50-100	≥1,000
Accuracy:	High if performed skillfully	High[a], reduced operator bias
Relative cost:	Low	Moderate

[a]Several thousand cells per second can be counted using the bead-based flow cytometry method. This makes it quick and accurate when dealing with many different samples. However, the reliability of the final counts depends on precise sample preparation, accurate cytometer performance, exactness of bead concentration, and the correctness of calculations. Bead lots come with a known concentration of beads per milliliter. If bead concentration is of concern, a hemacytometer can be used to verify bead concentration.

Table S8. HTS loader settings regularly used for the analysis of the cytotoxicity assay outlined in this protocol, related to Step 5 of "Analysis of cytotoxicity assay".

Sample flow rate (µL/second)	Sample volume (µL)	Mixing volume (µL)	Mixing speed (µL/second)	Number of mixes	Wash volume (µL)	Enable BLR	BLR period
3.0	40	40	200	3	400	Yes	5
